# Ophiostomatoid fungi associated with conifer-infesting beetles and their phoretic mites in Yunnan, China

**DOI:** 10.3897/mycokeys.28.21758

**Published:** 2017-12-21

**Authors:** Runlei Chang, Tuan A. Duong, Stephen J. Taerum, Michael J. Wingfield, Xudong Zhou, Z. Wilhelm de Beer

**Affiliations:** 1 Department of Microbiology and Plant Pathology, Forestry and Agricultural Biotechnology Institute (FABI), University of Pretoria, Pretoria 0002, South Africa; 2 Department of Genetics, Forestry and Agricultural Biotechnology Institute (FABI), University of Pretoria, Pretoria 0002, South Africa; 3 FuturaGene Biotechnology (Shanghai) Co., Ltd., Xuhui, Shanghai 200235, China

**Keywords:** Symbiont, species diversity, fungal vector, *Ophiostoma quercus*

## Abstract

The Ophiostomatales is an Ascomycete order of fungi that accommodates several tree pathogens and many species that degrade wood. These fungi are commonly vectored by Scolytine bark and ambrosia beetles. In recent years it has also been shown that hyperphoretic mites on these beetles can vector some Ophiostomatales. Little is known regarding the Ophiostomatales in China and we have consequently explored the diversity of these fungi associated with conifer-infesting beetles and mites in Yunnan province. Galleries and beetles were collected for 17 beetle species, while 13 mite species were obtained from six of these beetle species. Collectively, 340 fungal isolates were obtained, 45 from beetles, 184 from mites, 56 from galleries and 55 isolates where the specific niche was not clear. DNA sequences for five gene regions (ITS, LSU, BT, EF, and CAL) were determined for fungal isolates representing different morphological groups. Phylogenetic analyses confirmed the presence of 19 fungal taxa, including five novel species described here as *Ophiostoma acarorum* **sp. nov.**, *Ophiostoma brevipilosi* **sp. nov.**, *Graphilbum kesiyae* **sp. nov.**, *Graphilbum puerense* **sp. nov.**, and *Leptographium ningerense* **sp. nov.** *Ophiostoma ips* was the most frequently isolated species, representing approximately 31% of all isolates. Six of 19 taxa were present on mites, beetles and in the galleries of the beetles, while three species were found on mites and galleries. Two species were found only on mites and one species only on a beetle. Although the numbers of beetles and mites were insufficient to provide statistical inferences, this study confirmed that mites are important vectors of the Ophiostomatales in China. We hypothesize that these mites are most likely responsible for horizontal transfer of fungal species between galleries of different beetle species. The fact that half of the fungal species found were new to science, suggests that the forests of east Asia include many undescribed Ophiostomatales yet to be discovered.

## Introduction

The ophiostomatoid fungi represent a polyphyletic group of tree- or wood-infecting fungi, most often staining freshly exposed sapwood and thus lowering the value of timber (Seifert 1993). Some of the species are important tree pathogens that have dramatically impacted natural forests and caused major losses to forestry companies during the course of the last century (Harrington 1993, Wingfield et al. 2017). The sexual and often asexual spore-bearing structures of the ophiostomatoid fungi are specifically adapted for dispersal by arthropods, producing spores in sticky droplets that attach to the bodies of their vectors (Malloch and Blackwell 1993). However, these morphological and biological traits have evolved more than once in the Ascomycota and phylogenetic analyses have shown that these fungi reside in two different orders, the Ophiostomatales and Microascales (De Beer et al. 2013). The Ophiostomatales currently accommodates a single family, the Ophiostomataceae, that includes ten genera (Bateman et al. 2017, De Beer et al. 2016a, De Beer et al. 2016b, Van der Linde et al. 2016), while the Microascales comprises of five families, of which the Ceratocystidaceae, Gondwanamycetaceae and Graphiaceae include 14 genera of ophiostomatoid fungi (De Beer et al. 2014, 2017, Mayers et al. 2015, Nel et al. 2017).

Bark beetles (Coleoptera: Scolytinae) are well known vectors of ophiostomatoid fungi (Harrington 2005, Kirisits 2004, Six 2003). Other than a few bark beetle species that are considered to be primary pests causing significant economic losses, most species of bark beetles are secondary invaders, which colonize injured or stressed trees (Harrington 2005, Six and Wingfield 2011). Some bark beetle species have specialized structures known as mycangia that carry fungal conidia or ascospores, but most of these insects carry fungi on their exoskeletons (Six 2003). They construct their galleries, where the ophiostomatoid fungi grow, in the phloem and cambial layers under the bark of trees (Paine et al. 1997). The nature of the relationships between fungi and bark beetles is recognized as being very diverse. Some beetle-fungus symbioses appear to be obligate, while others may be facultative (Kirisits 2004). The role of the fungi can vary from their being nutritional symbionts of the beetles to saprophytes in the galleries, although in most cases the exact nature of the symbiosis is not well understood (Six and Wingfield 2011).

Along with the fungi, mites are also common symbionts of bark beetles (Hofstetter and Moser 2014, Roets et al. 2009). Due to their small body size and low mobility, many mite species rely on beetles or other animals for dispersal (Mitchell 1970). The relationship between mites and their vectors is usually commensal, but in some cases these are antagonistic or even mutualistic (Pfammatter et al. 2013). The phoretic mites of only a limited number of bark beetles have been studied in detail, including mostly North American species of *Scolytus*, *Dendroctonus* and *Ips* (Hofstetter et al. 2013).

As is the case with bark beetles, their mite associates can also vector ophiostomatoid fungi (Hofstetter and Moser 2014). Some mites such as *Tarsonemus* spp. possess sporothecae that are specialized structures of the integument that serve to carry fungal spores (Hofstetter et al. 2015). Ophiostomatoid fungi associated with mites include genera such as *Ceratocystis*, *Knoxdaviesia*, *Ceratocystiopsis*, *Ophiostoma*, *Leptographium*, *Grosmannia* and *Sporothrix* (Hofstetter et al. 2013). The interactions between mites and fungi are dynamic and vary with tree host condition and climate (Klepzig and Hofstetter 2011). It is for example known that mites can promote some fungal growth by increasing fungal transmission, reproduction, and survival, or by reducing the abundance of antagonistic fungi (Hofstetter and Moser 2014). Mites can also negatively affect some fungi because of exploitative/interference competition, predation, or encouragement of antagonistic fungi (Hofstetter et al. 2006).

Research on the interactions between ophiostomatoid fungi and bark beetles have been ongoing for more than a century in North America (Harrington 2005, Hedgcock 1906), Europe (Kirisits 2004, Münch 1907), and Japan (Masuya et al. 2013, Nisikado and Yamauti 1933). The associations of ophiostomatoid fungi with mites have been studied only in North America and Europe (Hofstetter and Moser 2014, Hofstetter et al. 2013), with a single report from Japan (Moser et al. 1997) and with ongoing studies on *Protea* spp. in Southern Africa (Roets et al. 2009, Roets et al. 2007). It is only more recently that the fungal associates of bark beetles have been explored in China, despite the fact that more than 160 bark beetle species have been recorded from that country (Yin et al. 1984).

The first new beetle associated ophiostomatoid species described from China for which the description was supported by DNA sequence data, was *Grosmannia yunnanensis* (Zhou et al. 2000, Yamaoka et al. 2008). Additional new species described during the past decade from a variety of conifer-infesting beetles in China, include one *Graphium* (Paciura et al. 2010a), 12 *Leptographium* (Liu et al. 2017, Lu et al. 2008, Paciura et al. 2010b, Yin et al. 2015), and five *Ophiostoma* (Yin et al. 2016) species. The most comprehensive study on the fungal associates of a specific beetle species in China has been on *Dendroctonus valens*, an economically important invasive bark beetle that was introduced to China from the USA and is attacking native pine species (Lu et al. 2008, 2009a, b, Taerum et al. 2013). Together with the fungal associates of *D. valens*, almost 40 ophiostomatoid species have been recorded and described from conifers in China (Liu et al. 2017, Paciura et al. 2010a, b, Yin et al. 2015, 2016, Zhou et al. 2013). Most of these are from bark beetles or their galleries. The fungal associates of mites have not been studied in this country (Hofstetter et al. 2013, Zhou et al. 2013).

Yunnan province forms part of in the southwestern forest zone, the second largest forest area in China (http://www.china.org.cn/english/shuzi-en/en-shuzi/gq/htm/zrzy-land-sl.htm). This province has a unique geography where three climatic regions meet: the eastern Asia monsoon region, the Tibetan plateau region, and the tropical monsoon region of southern Asia and Indo-China. As a result , species diversity in Yunnan province is high when compared to other parts of China. For example, more than 18000 plant species and 1836 vertebrate species are found here which represent 51.6% and 54.8% respectively of total species numbers in China (Yang et al. 2004). However, only 13 ophiostomatoid species have been reported from Yunnan (Zhou et al. 2013). Based on the high diversity of trees and vertebrates, but the relatively low number of ophiostomatoid species reported from Yunnan to date, we hypothesize that there are many undiscovered species from this group of fungi associated with bark beetles and mites in this province. The aim of this study was thus to further explore the diversity of ophiostomatoid fungi associated with conifer-infesting beetles and mites in Yunnan.

## Material and methods

### Collection of bark beetles and mites

Three surveys were conducted in Yunnan during the flight period of bark beetles in July 2001, July 2002, and between June and September 2010 (Table 1, Suppl. material 2: Figure S1). Surveys were conducted both in sawmill log-yards and forest areas. During the 2001 and 2002 surveys, only galleries and beetles were considered from pine species in conifer and mixed forests in central and northwestern Yunnan (Table 1, Suppl. material 2: Figure S1). During the 2010 survey, some collections were conducted using *Pinus kesiya* log traps in the pine forests around Pu’er city in southwestern Yunnan. Both adult bark beetles and their galleries were collected and stored in Eppendorf tubes and re-sealable bags, respectively. Mites were carefully removed from these beetles and galleries in the laboratory and individually placed in Eppendorf tubes. After the isolation of fungi, beetles and mites were stored in 75% ethanol for later identification. Beetles and mites were identified based on the morphology by Dr R. Beaver (Chiang Mai University, Thailand) and Dr E.A. Ueckermann (Plant Protection Research Institute, Agricultural Research Council, South Africa) respectively.

**Table 1.** Conifer-infesting bark and ambrosia beetles (Scolytinae), weevils (Molytinae), true weevils (Cossoninae), cylindrical bark beetles (Colydiinae), and mites (Acari) collected from Yunnan in this study. Numbers in the table refer to number of mite individuals collected.

Beetle species		Family	Subfamily	Tree host	Origin	Collection dates	Mite species													
							a	b	c	d	e	f	g	h	i	j	k	l	m	Total
A	*Coccotrypes cyperi*	Curculionidae	Scolytinae	*Pinus kesiya*	Ning’er	Sep. 2010	16	3	9	0	0	0	2	4	0	0	0	0	3	37
B	*Cyrtogenius luteus*	Curculionidae	Scolytinae	*P. kesiya*	Ning’er	Sep. 2010	0	2	10	0	0	0	0	0	0	0	0	0	0	12
C	*Hylurgops major*	Curculionidae	Scolytinae	*Pinus yunnanensis*	Zixishan	Jul. 2002	0	0	0	0	0	0	0	0	0	0	0	0	0	0
D	*Ips acuminatus*	Curculionidae	Scolytinae	*P. kesiya*	Ning’er, Puer	Jun. 2010	0	0	0	6	0	0	0	0	0	0	0	0	2	8
E	*Lasconotus* sp.	Zopheridae	Colydiinae	*P. kesiya*	Ning’er	Jun. 2010	0	0	0	0	0	0	0	0	0	0	0	0	0	0
F	*Orthotomicus angulatus*	Curculionidae	Scolytinae	*P. kesiya*	Ning’er	Sep. 2010	0	0	4	13	0	0	0	0	1	0	0	1	0	19
G	*Pissodes* sp.	Curculionidae	Molytinae	*Pinus armandii*	Lijiang, Midu	Jul. 2001	0	0	0	0	0	0	0	0	0	0	0	0	0	0
H	*Polygraphus aterrimus*	Curculionidae	Scolytinae	*P. kesiya*	Ning’er	Jun. 2010	0	0	0	0	0	0	0	0	0	0	0	0	0	0
I	*Polygraphus* sp.	Curculionidae	Scolytinae	*P. kesiya*	Ning’er	Jun. 2010	0	0	0	6	0	4	0	0	0	0	0	0	0	10
J	*P. szemaoensis*	Curculionidae	Scolytinae	*P. kesiya*	Ning’er, Simao	Jun. 2010	0	0	0	14	1	0	0	0	0	2	2	0	1	20
K	*P. verrucifrons*	Curculionidae	Scolytinae	*P. yunnanensis*	Lufeng	Jul. 2002	0	0	0	0	0	0	0	0	0	0	0	0	0	0
L	*Stenoscelis* sp.	Curculionidae	Cossoninae	*P. kesiya*	Ning’er	Jun. 2010	0	0	0	0	0	0	0	0	0	0	0	0	0	0
M	*Tomicus minor*	Curculionidae	Scolytinae	*P. yunnanensis*	Zixishan	Jul. 2002	0	0	0	0	0	0	0	0	0	0	0	0	0	0
N	*T. piniperda*	Curculionidae	Scolytinae	*P. yunnanensis*	Lufeng, Zixishan, Changhu	Jul. 2002	0	0	0	0	0	0	0	0	0	0	0	0	0	0
O	*T. brevipilosus*	Curculionidae	Scolytinae	*P. kesiya*	Ning’er	Jun. 2010	0	0	0	0	0	0	0	0	0	0	0	0	0	0
P	Unknown sp.1	Curculionidae	Scolytinae	*P. semaonensis*	Chuxiong	Jul. 2002	0	0	0	0	0	0	0	0	0	0	0	0	0	0
Q	Unknown sp.2	Curculionidae	Molytinae	*Tsuga* sp.	Dali	Jul. 2002	0	0	0	0	0	0	0	0	0	0	0	0	0	0
**Total**							**16**	**5**	**23**	**39**	**1**	**4**	**2**	**4**	**1**	**2**	**2**	**1**	**6**	**106**

Mite species: a = *Dendrolaelaps* sp. 1 (Mesostigmata, Digamasellidae); b = *Dendrolaelaps* sp. 2 (Mesostigmata, Digamasellidae); c = *Histiostoma* cf. *sapromyzarum* (Sarcoptiformes, Histiostomatidae); d = *Insectolaelaps* sp. 1 (Mesostigmata, Digamasellidae); e = *Lasioseius* sp. 1 (Mesostigmata, Blattisociidae); f = *Proctolaelaps nr. hystrix* (Mesostigmata, Melicharidae); g = *Rhizoglyphus* sp. 1 (Sarcoptiformes, Acaridae); h = *Schwiebea taiwanensis* (Sarcoptiformes, Acaridae); I = Unknown sp.1 (Mesostigmata); j = Unknown sp.2 (Oribatei); k = Unknown sp.3 (Mesostigmata, Uropodoidea); l = Unknown sp.4 (Mesostigmata); m = Unknown sp.5.

### Isolation of fungi

Fungi were isolated from the bark beetles and their galleries following the methods described by Linnakoski et al. (2008). At the time of collection, living mites collected from the galleries were placed on the surface of 2% MEA medium (20 g Difco agar, 20 g Difco BactoTM malt extract [Becton, Dickinson & Company], 1 L deionized water) and allowed to crawl over the plate for 24 hours. The mites were then removed and stored in Eppendorf tubes for identification. MEA plates were monitored daily for fungal growth and hyphal tips of emerging colonies were transferred to fresh MEA plates in order to purify the fungi. Pure cultures were grouped based on culture morphology, and representatives from each group were selected for DNA sequencing. All isolates used in this study were deposited in the Culture Collection (CMW) of the Forestry and Agricultural Biotechnology Institute (FABI), University of Pretoria, Pretoria, Republic of South Africa. Isolates representing types of new species were also deposited in the culture collection (CBS) of the Westerdijk Fungal Biodiversity Institute, Utrecht, the Netherlands.

### DNA sequencing and phylogenetic analyses

Isolates were grown on 2% MEA in Petri dishes. DNA was extracted using PrepMan Ultra Sample Preparation reagent (Applied Biosystems, Foster City, CA) following the manufacturer’s instructions. The primer pairs ITS1F (Gardes and Bruns 1993) and ITS4 (White et al. 1990) were used to amplify internal transcribed spacer regions (ITS), the primary barcode of fungi (Schoch et al. 2012). LR0R and LR5 (Vilgalys and Hester 1990) were used to amplify nuclear large subunit (LSU). For ITS2-LSU used in *Leptographium*, ITS and LSU sequences were combined. EF2F (Marincowitz et al. 2015) and EF2R (Jacobs et al. 2004) were used to amplify the elongation factor 1-α (EF) gene, the secondary barcode of fungi (Stielow et al. 2015). BT2A and BT2B (Glass and Donaldson 1995) were used to amplify part of the β-tubulin (BT) gene, and CL2F and CL2R (Duong et al. 2012) for the calmodulin (CAL) gene. PCR and sequencing were conducted using the methods described by Duong et al. (2012).

The sequences obtained using the forward and reverse primers were aligned in Geneious Pro v. 7.1.4 (Biomatters, Auckland, New Zealand). ITS sequences were submitted to BLAST searches in NCBI Genbank for preliminary identifications. Based on these results, the ITS data were separated into different data sets according to genus. ITS2-LSU data were used for analyses of taxa residing in *Leptographium* *s.l.*, while ITS was used for analyses of taxa belonging to the other genera. The BT, EF and CAL data were separated into smaller data sets based on the species complexes as defined by De Beer and Wingfield (2013). All sequences obtained in this study were submitted to GenBank (Table 2). Alignments were done online with MAFFT v. 7 (Katoh and Standley 2013). Maximum likelihood (ML), maximum parsimony (MP) and Bayesian Inference (BI) were conducted on the datasets as described in Duong et al. (2012). For haplotype analyses of *O. quercus* and *O. tsotsi*, the online server ElimDupes was used (http://www.hiv.lanl.gov/content/sequence/ELIMDUPES/elimdupes.html).

**Table 2.** Isolates of ophiostomatoid fungi obtained from different beetles, their galleries and mites in Yunnan.

Taxon	Species	Isolate number^1,2^		Host	Location	Beetle	G/B/M^3^	GenBank number^4^			
		CMW	CBS					ITS/ITS2-LSU^5^	BT	EF	CAL
**Ophiostomatales**											
1	*Sporothrix* sp. A	41787		*Pinus kesiya*	Ning’er	*Coccotrypes cyperi*	M	MG205645	MG205681	–	–
2	*S. nebularis*	11791		*Pinus yunnanensis*	Zixishan	*Tomicus piniperda*	*	MG205646	MG205682	–	–
		41762		*P. kesiya*	Ning’er	*Co. cyperi*	M	MG205647	MG205683	–	–
		41776		*P. kesiya*	Ning’er	*Co. cyperi*	M	MG205648	MG205684	–	–
		41779		*P. kesiya*	Ning’er	*Co. cyperi*	M	MG205649	=MG205684	–	–
		41782		*P. kesiya*	Ning’er	*Co. cyperi*	M	MG205650	=MG205684	–	–
		41816		*P. kesiya*	Ning’er	*Cyrtogenius luteus*	M	MG205651	MG205685	–	–
		41819		*P. kesiya*	Ning’er	*Orthotomicus angulatus*	M	MG205652	=MG205685	–	–
		41835		*P. kesiya*	Ning’er	*Polygraphus* sp.	M	MG205653	=MG205684	–	–
3	*Ophiostoma acarorum* sp. nov.	41630		*P. kesiya*	Ning’er	*Ips acuminatus*	G	=MG205656	MG205686	–	–
		41641		*P. kesiya*	Ning’er	*Polygraphus szemaoensis*	G	=MG205656	=MG205686	–	–
		41642		*P. kesiya*	Ning’er	*P. szemaoensis*	G	=MG205656	=MG205686	–	–
		41647		*P. kesiya*	Pu’er	*I. acuminatus*	M	=MG205656	=MG205686	–	–
		41789		*P. kesiya*	Ning’er	*Cy. luteus*	M	=MG205657	=MG205686	–	–
		41791		*P. kesiya*	Ning’er	*Cy. luteus*	M	=MG205657	=MG205686	–	–
		41795		*P. kesiya*	Ning’er	*Cy. luteus*	M	=MG205657	=MG205686	–	–
		41798	139643	*P. kesiya*	Ning’er	*Cy. luteus*	M	=MG205657	=MG205686	–	–
		41812	139658	*P. kesiya*	Ning’er	*Cy. luteus*	M	MG205656	=MG205686	–	–
		41850^H^	139748	*P. kesiya*	Ning’er	*O. angulatus*	M	MG205657	=MG205686	–	–
		41852		*P. kesiya*	Ning’er	*Co. cyperi*	M	=MG205657	=MG205686	–	–
		41987		*P. kesiya*	Ning’er	*I. acuminatus*	G	=MG205656	=MG205686	–	–
4	*O. ips*	41620		*P. kesiya*	Ning’er	*I. acuminatus*	G	MG205658	MG205687	–	–
		41644		*P. kesiya*	Pu’er	*I. acuminatus*	B	=MG205658	=MG205687	–	–
4	*O. ips*	41653		*P. kesiya*	Ning’er	*P. szemaoensis*	G	=MG205658	MG205688	–	–
		41695		*P. kesiya*	Simao	*P. szemaoensis*	M	=MG205658	=MG205688	–	–
		41697		*P. kesiya*	Simao	*P. szemaoensis*	B	=MG205658	=MG205688	–	–
		41709		*P. kesiya*	Simao	*P. szemaoensis*	B	=MG205658	=MG205688	–	–
		41745		*P. kesiya*	Ning’er	*Polygraphus* sp.	M	=MG205658	=MG205688	–	–
		41916		*P. kesiya*	Ning’er	*P. szemaoensis*	G	=MG205658	=MG205688	–	–
		41993		*P. kesiya*	Ning’er	*I. acuminatus*	M	=MG205658	=MG205687	–	–
5	*Ophiostoma* sp. B	12032		*Pinus semaonensis*	Chuxiong	Unknown sp.1	*	MG205659	MG205689	MG205731	–
6	*O. brevipilosi* sp. nov.	41624	139661	*P. kesiya*	Ning’er	*Tomicus brevipilosus*	B	=MG205660	MG205690	MG205732	–
		41662^H^	139659	*P. kesiya*	Ning’er	*T. brevipilosus*	B	MG205660	=MG205690	=MG205732	–
		41760		*P. kesiya*	Ning’er	*T. brevipilosus*	B	=MG205660	=MG205690	=MG205732	–
		41873	139660	*P. kesiya*	Ning’er	*T. brevipilosus*	B	=MG205660	=MG205690	=MG205732	–
		41932		*P. kesiya*	Ning’er	*T. brevipilosus*	B	=MG205660	=MG205690	=MG205732	–
		41995		*P. kesiya*	Ning’er	*T. brevipilosus*	B	=MG205660	=MG205690	=MG205732	–
7	*O. setosum*	12152		*Tsuga* sp.	Dali	Unknown sp.2	*	MG205661	MG205691	MG205733	–
		12192		*Tsuga* sp.	Dali	Unknown sp.2	*	=MG205661	=MG205691	MG205734	–
		12337		*Tsuga* sp.	Dali	Unknown sp.2	*	=MG205661	=MG205691	MG205735	–
8	*O. quercus*	11747		*P. yunnanensis*	Lufeng	*Polygraphus verrucifrons*	*	=AF198238	=FJ455565	MG205736	–
		11748		*P. yunnanensis*	Lufeng	*P. verrucifrons*	*	=AF198238	=FJ455565	=MG205736	–
		11756		*P. yunnanensis*	Lufeng	*P. verrucifrons*	*	MG205662	MG205692	MG205737	–
		11806		*P. yunnanensis*	Changhu	*T. piniperda*	*	=FJ434947	=FJ455565	MG205738	–
		11807		*P. yunnanensis*	Changhu	*T. piniperda*	*	=FJ434947	=AY466647	=MG205738	–
		11981		*Abies* sp.	Chuxiong	Unknown sp.1	*	=FJ434947	=FJ455570	MG205739	–
		12015		*P. semaonensis*	Chuxiong	Unknown sp.1	*	=AY466624	MG205693	MG205740	–
		12037		Unknown	Chuxiong	Unknown sp.1	*	=AY466624	MG205694	MG205741	–
		12039		Unknown	Chuxiong	Unknown sp.1	*	=FJ434947	MG205695	MG205742	–
8	*O. quercus*	12122		*Tsuga* sp.	Dali	Unknown sp.2	*	=FJ434947	MG205696	MG205743	–
		12146		*Tsuga* sp.	Dali	Unknown sp.2	*	=FJ434947	=MG205696	=MG205743	–
		12185		*Tsuga* sp.	Dali	Unknown sp.2	*	=FJ434947	MG205697	=MG205740	–
		12195		*Tsuga* sp.	Dali	Unknown sp.2	*	=FJ434947	=MG205697	=MG205740	–
		12286		*Tsuga* sp.	Dali	Unknown sp.2	*	=FJ434947	=FJ455570	MG205744	–
		12350		*Tsuga* sp.	Dali	Unknown sp.2	*	=FJ434947	MG205698	MG205745	–
		12359		*Tsuga* sp.	Dali	Unknown sp.2	*	=AF198238	=FJ455570	MG205746	–
		12364		*Tsuga* sp.	Dali	Unknown sp.2	*	=AF198238	MG205699	MG214780	–
		12370		*Tsuga* sp.	Dali	Unknown sp.2	*	=AF198238	=MG205698	MG205747	–
		12382		*Tsuga* sp.	Dali	Unknown sp.2	*	=AF198238	=FJ455570	MG205748	–
		41659		*P. kesiya*	Pu’er	*I. acuminatus*	G	MG205664	MG205700	MG205749	–
		41693		*P. kesiya*	Simao	*P. szemaoensis*	G	=MG205664	=MG205700	=MG205749	–
		41715		*P. kesiya*	Simao	*P. szemaoensis*	B	=MG205664	=MG205700	=MG205749	–
		41718		*P. kesiya*	Simao	*P. szemaoensis*	B	=MG205664	=MG205700	=MG205749	–
		41724		*P. kesiya*	Ning’er	*P. szemaoensis*	B	MG205665	MG205701	MG205750	–
		41732		*P. kesiya*	Ning’er	*Cy. luteus*	G	=MG205664	=MG205700	=MG205749	–
9	*O. tsotsi*	41730		*P. kesiya*	Ning’er	*Co. cyperi*	G	=FJ441284	MG205704	MG205755	–
		41731		*P. kesiya*	Ning’er	*Co. cyperi*	G	=FJ441284	MG205705	MG205756	–
		41733		*P. kesiya*	Ning’er	*Cy. luteus*	G	=FJ441284	=MG205704	=MG205755	–
		41734		*P. kesiya*	Ning’er	*Cy. luteus*	G	=FJ441284	=MG205704	=MG205755	–
		41735		*P. kesiya*	Ning’er	*Co. cyperi*	M	=FJ441284	=MG205704	=MG205755	–
		41742		*P. kesiya*	Ning’er	*Co. cyperi*	M	=FJ441284	=MG205705	=MG205756	–
		41746		*P. kesiya*	Ning’er	*Co. cyperi*	M	=FJ441284	=MG205704	=MG205755	–
		41758		*P. kesiya*	Ning’er	*Co. cyperi*	M	=FJ441284	=MG205705	=MG205756	–
10	*Ophiostoma* sp. C	12150		*Tsuga* sp.	Dali	Unknown sp.2	*	MG205666	MG205709	MG205762	–
11	*Graphilbum fragrans*	11778		*P. yunnanensis*	Zixishan	*Tomicus minor*	*	MG205667	MG205710	–	–
12	*Gra. kesiyae* sp. nov.	41626		*P. kesiya*	Ning’er	*P. szemaoensis*	M	=MG205669	–	–	–
		41657	139639	*P. kesiya*	Ning’er	*Polygraphus* sp.	M	MG205668	=MG205714	–	–
12	*Gra. kesiyae* sp. nov.	41686	139641	*P. kesiya*	Simao	*P. szemaoensis*	M	=MG205668	MG205711	–	–
		41691	139642	*P. kesiya*	Simao	*P. szemaoensis*	M	=MG205669	=MG205713	–	–
		41703		*P. kesiya*	Simao	*P. szemaoensis*	G	–	MG205712	–	–
		41716	139657	*P. kesiya*	Simao	*P. szemaoensis*	M	=MG205669	=MG205712	–	–
		41729^H^	139652	*P. kesiya*	Ning’er	*P. szemaoensis*	G	MG205669	MG205713	–	–
		41774	139653	*P. kesiya*	Ning’er	*Polygraphus aterrimus*	B	MG205668	MG205714	–	–
		46468		*P. kesiya*	Ning’er	*P. aterrimus*	G	MG205668	=MG205714	–	–
		46469		*P. kesiya*	Ning’er	*P. aterrimus*	B	MG205668	=MG205714	–	–
13	*Gra. puerense* sp. nov.	41619		*P. kesiya*	Ning’er	*P. szemaoensis*	B	=MG205670	MG205715	–	–
		41667	139651	*P. kesiya*	Pu’er	*I. acuminatus*	G	MG205670	MG205716	–	–
		41670		*P. kesiya*	Ning’er	*I. acuminatus*	B	=MG205670	–	–	–
		41671		*P. kesiya*	Ning’er	*I. acuminatus*	B	=MG205670	MG205717	–	–
		41673	139640	*P. kesiya*	Ning’er	*I. acuminatus*	B	=MG205670	MG205718	–	–
		41942^H^	139650	*P. kesiya*	Ning’er	*P. szemaoensis*	G	MG205671	MG205719	–	–
		41971		*P. kesiya*	Ning’er	*P. szemaoensis*	M	=MG205671	MG205720	–	–
		41996		*P. kesiya*	Ning’er	*I. acuminatus*	M	=MG205671	MG205721	–	–
		41998		*P. kesiya*	Ning’er	*I. acuminatus*	G	=MG205670	=MG205715	–	–
14	*Leptographium gracile*	12305		*Pinus armandii*	Lijiang	*Pissodes* sp.	*	MG205672	MG205722	MG205763	MG205782
		12397		*P. armandii*	Midu	*Pissodes* sp.	*	=MG205672	=MG205722	=MG205763	=MG205782
		12399		*P. armandii*	Midu	*Pissodes* sp.	*	=MG205672	=MG205722	=MG205763	=MG205782
		12404		*P. armandii*	Midu	*Pissodes* sp.	*	=MG205672	=MG205722	=MG205763	=MG205782
		12407		*P. armandii*	Midu	*Pissodes* sp.	*	=MG205672	=MG205722	=MG205763	=MG205782
		12412		*P. armandii*	Midu	*Pissodes* sp.	*	=MG205672	=MG205722	=MG205763	=MG205782
15	*Grosmannia radiaticola*	12323		*Tsuga* sp.	Dali	Unknown sp.2	*	MG205673	MG205723	MG205764	–
16	*L. ningerense* sp. nov.	41773		*P. kesiya*	Ning’er	*Co. cyperi*	M	–	=MG205724	MG205765	=MG205783
		41786^H^	139663	*P. kesiya*	Ning’er	*Co. cyperi*	M	MG205674	MG205724	=MG205765	MG205783
		41831	139664	*P. kesiya*	Ning’er	*O. angulatus*	M	MG205675	=MG205724	=MG205765	=MG205783
17	*G. yunnanensis*	41622		*P. kesiya*	Ning’er	*P. szemaoensis*	B	MG205676	MG205725	MG205766	–
		41627		*P. kesiya*	Ning’er	*P. szemaoensis* or *I. acuminatus*	M	–	=MG205725	–	–
		41633		*P. kesiya*	Ning’er	*P. szemaoensis* or *I. acuminatus*	M	–	MG205726	–	–
		41635		*P. kesiya*	Ning’er	*Lasconotus* sp.	B	–	=MG205726	MG205767	–
		41636		*P. kesiya*	Ning’er	*Lasconotus* sp.	B	–	=MG205726	MG205768	–
		41666		*P. kesiya*	Ning’er	*Polygraphus* sp.	M	–	=MG205725	–	–
		41687		*P. kesiya*	Simao	*P. szemaoensis*	M	–	=MG205725	MG205769	–
		41694		*P. kesiya*	Simao	*P. szemaoensis*	M	–	=MG205725	–	–
		41707		*P. kesiya*	Simao	*P. szemaoensis*	B	–	=MG205725	MG205770	–
		41720		*P. kesiya*	Simao	*P. szemaoensis*	G	–	=MG205725	MG205771	–
		41721		*P. kesiya*	Ning’er	*P. szemaoensis*	M	–	=MG205725	MG205772	–
		41726		*P. kesiya*	Ning’er	*P. szemaoensis*	M	–	=MG205725	–	–
		41728		*P. kesiya*	Ning’er	*P. szemaoensis*	M	–	=MG205726	MG205773	–
		41777		*P. kesiya*	Ning’er	*Co. cyperi*	M	–	MG205727	MG205774	–
		41778		*P. kesiya*	Ning’er	*Co. cyperi*	M	–	MG205728	=MG205774	–
		41781		*P. kesiya*	Ning’er	*Co. cyperi*	M	–	=MG205727	=MG205774	–
		41783		*P. kesiya*	Ning’er	*Co. cyperi*	M	–	=MG205725	MG205775	–
		41805		*P. kesiya*	Ning’er	*Polygraphus* sp.	M	–	=MG205725	–	–
		41814		*P. kesiya*	Ning’er	*Polygraphus* sp.	M	–	=MG205725	–	–
		41858		*P. kesiya*	Ning’er	*P. szemaoensis*	G	–	=MG205725	MG205776	–
		41863		*P. kesiya*	Ning’er	*P. szemaoensis*	G	MG205677	=MG205725	MG205777	–
		41945		*P. kesiya*	Ning’er	*P. szemaoensis*	M	–	=MG205725	–	–
		41963		*P. kesiya*	Ning’er	*P. szemaoensis*	M	–	=MG205725	–	–
		41990		*P. kesiya*	Ning’er	*I. acuminatus*	G	–	=MG205725	=MG205776	–
17	*G. yunnanensis*	41992		*P. kesiya*	Ning’er	*I. acuminatus*	G	–	=MG205725	MG205778	–
		41999		*P. kesiya*	Ning’er	*P. szemaoensis*	B	–	=MG205725	–	–
		42000		*P. kesiya*	Ning’er	*P. szemaoensis*	B	–	–	=MG205778	–
18	*L. conjunctum*	11782		*P. yunnanensis*	Zixishan	*Hylurgops major*	*	MG205678	MG205729	MG205779	–
		41761		*P. kesiya*	Ning’er	*Polygraphus* sp.	M	MG205679	MG205730	MG205780	–
**Microascales**											
19	*Graphium pseudormiticum*	41665		*P. kesiya*	Pu’er	*I. acuminatus*	M	MG205680	–	MG205781	–

^1^ The culture collection (CBS) of Westerdijk Fungal Biodiversity Institute, Utrecht, the Netherlands; CMW Culture Collection of the Forestry and Agricultural Biotechnology Institute (FABI), University of Pretoria, Pretoria, South Africa ^2^ H = ex–holotype isolate ^3^ G = Gallery; B = Beetle; M = Mite; * = Unknown ^4^ ITS = internal transcribed spacer regions 1 and 2 of the nuclear ribosomal DNA operon, including the 5.8S region; ITS2–LSU = the internal transcribed spacer 2 region and partial large subunit of the nrDNA operon; ΒT = beta–tubulin; EF = translation elongation factor 1–alpha; CAL = Calmodulin

### Growth studies

Growth studies were conducted on three isolates of each novel taxon. Mycelium-covered agar plugs were transferred from the actively growing margins of one-week-old cultures and placed at the centers of 90 mm Petri dishes containing 2% MEA. Cultures were incubated in the dark at temperatures ranging from 5–35 °C at 5 °C intervals. Three replicates were used for each isolate at each temperature. Colony diameters were measured every day until hyphae reached the edges of the Petri dishes. Optimum and maximum growth temperatures were calculated for each species.

### Morphological studies

To facilitate morphological descriptions of new taxa, asexual and sexual structures (where present) were mounted in lactophenol on glass slides, covered with a coverslip and examined with a Zeiss Axioskop2 Plus compound microscope or a Zeiss Discovery V12 dissection microscope with an Axiocam digital camera (Axiovision 3.1) (München-Hallbergmoos, Germany). Measurements were made for each taxonomically characteristic structure. The measurements are presented in the format (minimum–) mean minus standard deviation–mean plus standard deviation (–maximum). For reference to asexual states that resemble morphological features of well-known asexual genera, we followed the reference style (e.g. hyalorhinocladiella-like) suggested by Hawksworth (2011). Descriptions of morphological features of *Leptographium* species were based on the style of Jacobs and Wingfield (2001).

### Frequency of isolation

Frequencies of isolation of the ophiostomatoid species in all samples were calculated as follows: F = (NF/NT) × 100, where F represents the frequency of isolation (%), NT represents the total number of isolates collected, and NF represents the number of isolates of a particular fungal species.

## Results

### Bark beetles and mites

Collectively, 17 beetle species belonging to four sub-families were collected from conifer hosts at 10 sites (Table 1). Seven of the species were collected in 2001 and 2002 and ten species in 2010. In total, 106 mites were collected from galleries of six Scolytine bark beetle species during the 2010 survey. One hundred of these mites represented 12 mite species belonging to eight families (Table 1). The remaining six mites (Table 1, Column m) could not be identified. The abundance of each mite species collected varied considerably, with *Insectolaelaps* sp. 1, Histiostoma cf. sapromyzarum and *Dendrolaelaps* sp. 1 being the most abundant overall. The other species were present in very low numbers. Among the most abundant mites, *Insectolaelaps* sp. 1 was found on four beetle species, and Histiostoma cf. sapromyzarum was found on three beetle species, while *Dendrolaelaps* sp. 1 was associated with only one beetle species. *Coccotrypes cyperi* was the most common beetle vector as it was phoreticed with 34 mites representing eight species, including three unknown species. Nineteen mites representing five species (including one unknown) came from *Polygraphus szemaoensis*, and *Orthotomicus angulatus* vectored 19 mites representing four species.

### Fungal isolation

A total of 340 fungal isolates were obtained, 54 from beetles in 2001 and 2002, and 286 isolated from beetles, galleries and mites in 2010 (Tables 3 and Suppl. material 1: Table S1). Bark beetle niches in this study include the beetles themselves, their galleries or the mites associated with a particular bark beetle or its galleries. The numbers of fungal isolates collected from the different bark beetle niches varied substantially. For example, 85 isolates were collected from the *P. szemaoensis* niche, 60 isolates from that of *C. cyperi*, but only two and four isolates from the niches of *Lasconotus* sp. and *P. aterrimus* respectively.

### DNA sequencing and phylogenetic analyses

From the total of 340 isolates obtained in the study, DNA sequences were generated for 134 isolates (Table 2), representing all the morphological groups. Based on preliminary BLAST results, ITS data generated in the present study were separated into two data sets, the first including *Ophiostoma*, *Sporothrix* and *Graphilbum* in the Ophiostomatales, and the second including *Graphium* spp. in the Microascales. Because amplification of the ITS1 and 2 regions is often problematic for *Leptographium* *s.l.* species (De Beer and Wingfield 2013), a reference data set consisting of ITS2-LSU data was compiled to determine the species complexes in *Leptographium* *s.l.* to which the isolates from China belonged.

**Figure 1.**
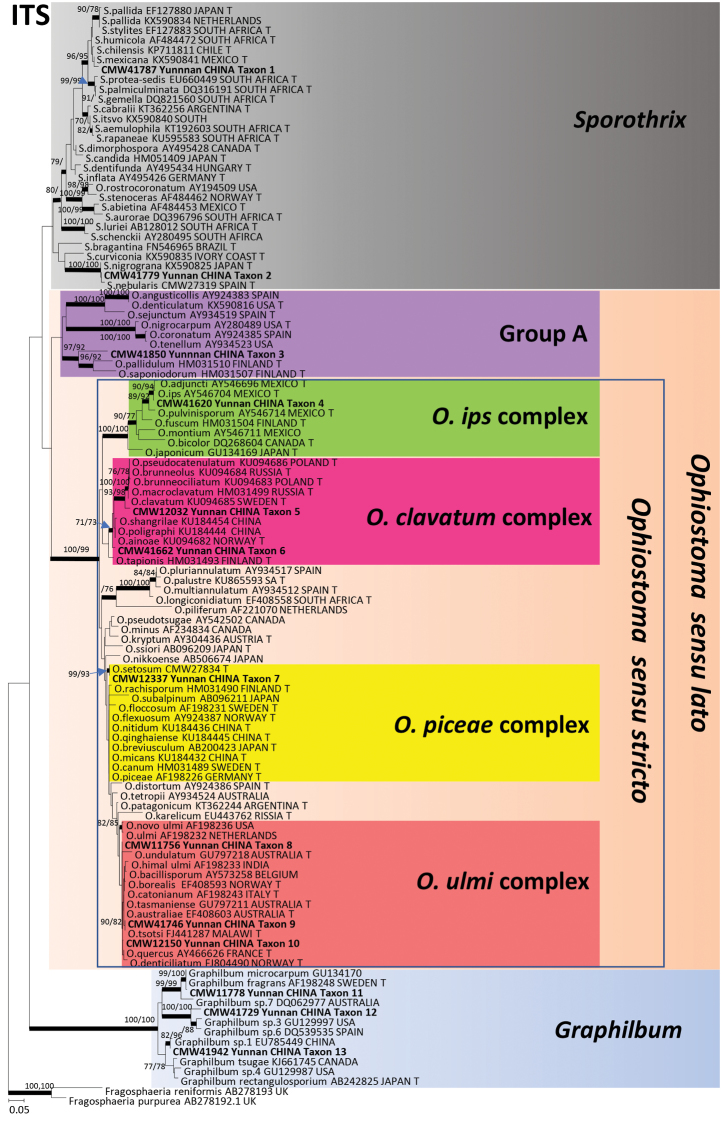
ML tree of the ITS region of *Ophiostoma*, *Sporothrix*, *Graphilbum*. Novel sequences obtained in this study are printed in **bold** type. *Bold* branches indicate posterior probabilities values ≥ 0.95. Bootstrap values ≥ 70 % are recorded at nodes as ML/MP. T = ex-type isolates.

**Figure 2.**
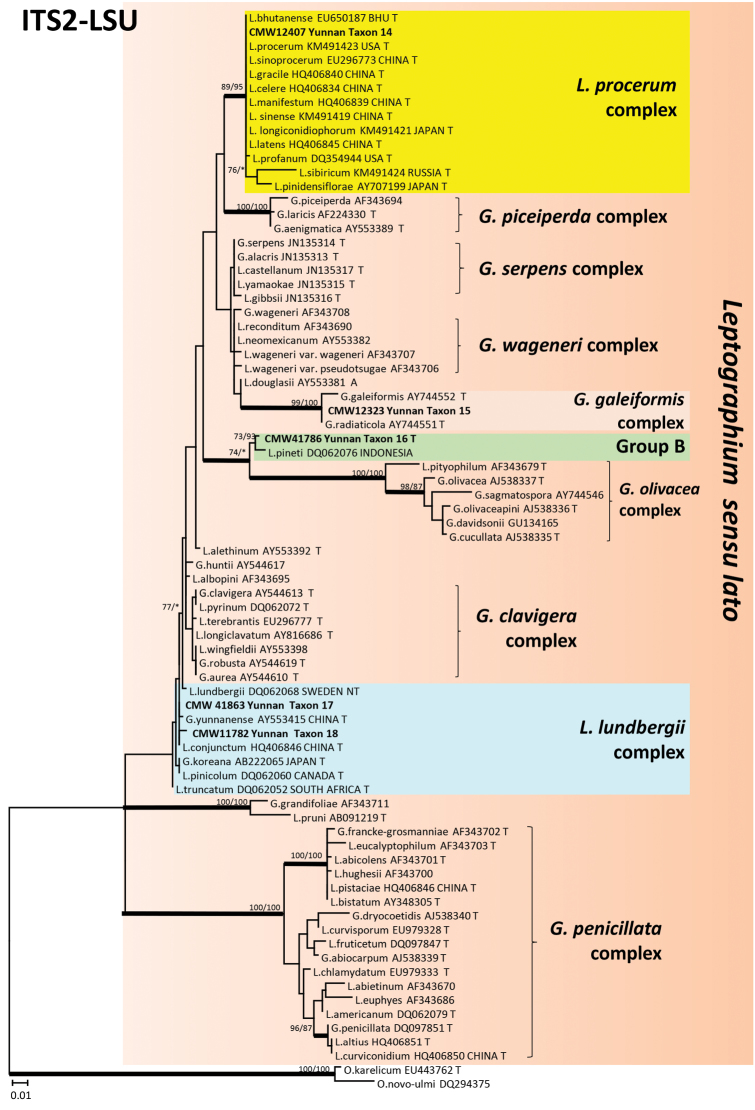
ML tree of the ITS2-LSU region of *Leptographium*. *Bold* branches indicate posterior probabilities values ≥ 0.95. Bootstrap values ≥ 70 % are recorded at nodes as ML/MP. T = ex-type isolates.

The 340 isolates were separated in 19 taxa (Taxa 1 to 19) based on DNA sequences (Table 2) and culture morphology. Taxa 1 and 2 (Fig. 1) represented species of *Sporothrix*, Taxa 3 to 10 resided in five species complexes in *Ophiostoma* *s.l.*, and Taxa 11 to 13 belonged to *Graphilbum*. Taxa 14 to 18 belonged to four species complexes in *Leptographium* *s.l.* (Fig. 2). One taxon (Taxon 19) resided in the genus *Graphium* (Suppl. material 2: Figure S1).

Two species of *Sporothrix* were collected (Suppl. material 2: Figure S3). Taxon 1 was represented by a single isolate and both ITS and BT data suggested that this was of an undescribed species. Taxon 2 included 45 isolates, eight for which sequence data were included in our analyses (Suppl. material 2: Figure S3). In the ITS tree, these isolates grouped in a monophyletic clade that included the ex-type isolates of *S. nebularis* and *S. nigrograna*. In the BT tree these isolates again grouped with *S. nebularis*.

**Figure 3.**
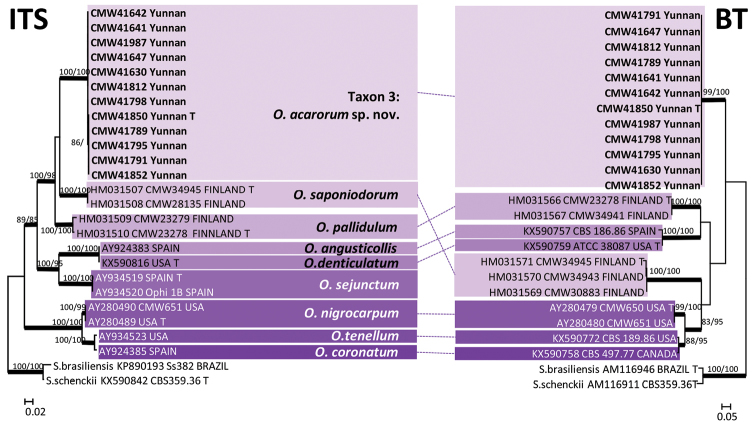
ML trees of Group A generated from DNA sequences of ITS and BT regions. *Bold* branches indicate posterior probabilities values ≥ 0.95. Bootstrap values ≥ 70 % are recorded at nodes as ML/MP. T = ex-type isolates.

Taxon 3 included 29 isolates (Table 3), 12 of which (Table 2) were included in the analyses. These isolates grouped with a number of *Ophiostoma* spp. peripheral to the well-defined complexes in *Ophiostoma* *s.str.*, and they were treated as Group A in *Ophiostoma* *s.l.* (Fig. 1). In both the ITS and BT trees (Fig. 3), the Yunnan isolates formed a well-supported lineage, closest to but clearly distinct from *O. pallidulum* and *O. saponiodorum*.

**Table 3.** Numbers of fungal isolates per species obtained from beetles (**B**), their galleries (**G**) or mite (**M**) associates

**Year of survey →**	**2010 **																														**2001**	**2002**						
**Host tree species^1 ^→**	**I**																														**II**	**III**				**IV**	**V**	**Total**
**Beetle species^2 ^→**	**A**			**B**			**D**			**E**			**F**			**H**			**I**			**J**			**L**			**O**			**G**	**C**	**K**	**M**	**N**	**P**	**Q**	
	**B**	**G**	**M**	**B**	**G**	**M**	**B**	**G**	**M**	**B**	**G**	**M**	**B**	**G**	**M**	**B**	**G**	**M**	**B**	**G**	**M**	**B**	**G**	**M**	**B**	**G**	**M**	**B**	**G**	**M**	*	*	*	*	*	*	*	
**Taxon. Fungus species↓**																																						
1. *Sporothrix* sp. A	0	0	0	0	0	1	0	0	0	0	0	0	0	0	0	0	0	0	0	0	0	0	0	0	0	0	0	0	0	0	0	0	0	0	0	0	0	1
2. *S. nebularis*	0	0	4	0	0	7	0	1	0	0	0	0	0	0	18	0	0	0	0	0	5	1	3	2	1	0	0	1	0	0	0	0	0	0	1	1	0	45
3. *Ophiostoma acarorum* sp. nov.	0	0	21	0	0	2	0	2	1	0	0	0	0	0	1	0	0	0	0	0	0	0	2	0	0	0	0	0	0	0	0	0	0	0	0	0	0	29
4. *O. ips*	0	0	1	0	1	17	6	11	14	0	0	0	0	0	13	0	1	0	0	0	2	9	15	5	0	0	0	0	0	0	0	0	3	0	4	4	0	106
5. *Ophiostoma* sp. B	0	0	0	0	0	0	0	0	0	0	0	0	0	0	0	0	0	0	0	0	0	0	0	0	0	0	0	0	0	0	0	0	0	0	0	1	0	1
6. *O. brevipilosi* sp. nov.	0	0	0	0	0	0	0	0	0	0	0	0	0	0	0	0	0	0	0	0	0	0	0	0	0	0	0	8	0	0	0	0	0	0	0	0	0	8
7. *O. setosum*	0	0	0	0	0	0	0	0	0	0	0	0	0	0	0	0	0	0	0	0	0	0	0	0	0	0	0	0	0	0	0	0	0	0	0	0	5	5
8. *O. quercus*	0	1	0	0	1	7	0	1	0	0	0	0	0	0	0	0	0	0	0	0	0	6	1	6	0	0	0	0	0	0	0	0	3	0	2	4	11	43
9. *O. tsotsi*	0	2	0	0	2	8	0	0	0	0	0	0	0	0	0	0	0	0	0	0	0	0	0	0	0	0	0	0	0	0	0	0	0	0	0	0	0	12
10. *Ophiostoma* sp. C	0	0	0	0	0	0	0	0	0	0	0	0	0	0	0	0	0	0	0	0	0	0	0	0	0	0	0	0	0	0	0	0	0	0	0	0	1	1
11. *Graphilbum fragrans*	0	0	0	0	0	0	0	0	0	0	0	0	0	0	0	0	0	0	0	0	0	0	0	0	0	0	0	0	0	0	0	0	0	1	0	0	0	1
12. *Gra. kesiyae* sp. nov.	0	0	0	0	0	0	0	0	0	0	0	0	0	0	0	2	1	0	0	0	2	0	2	5	0	0	0	0	0	0	0	0	0	0	0	0	0	11
13. *Gra. puerense* sp. nov.	0	0	0	0	0	0	4	2	2	0	0	0	0	0	0	0	0	0	0	0	0	1	1	1	0	0	0	0	0	0	0	0	0	0	0	0	0	11
14. *Leptographium gracile*	0	0	0	0	0	0	0	0	0	0	0	0	0	0	0	0	0	0	0	0	0	0	0	0	0	0	0	0	0	0	10	0	0	0	0	0	0	10
15. *G. radiaticola*	0	0	0	0	0	0	0	0	0	0	0	0	0	0	0	0	0	0	0	0	0	0	0	0	0	0	0	0	0	0	0	0	0	0	0	1	0	1
16. *L. ningerense* sp. nov.	0	0	2	0	0	8	0	0	0	0	0	0	0	0	2	0	0	0	0	0	0	0	1	0	0	0	0	0	0	0	0	0	0	0	0	0	0	13
17. *G. yunnanensis*	0	0	0	0	0	6	0	2	0	2	0	0	0	0	0	0	0	0	0	0	3	4	3	17	0	0	0	0	0	0	0	0	0	0	1	1	0	39
18. *L. conjunctum*	0	0	0	0	0	0	0	0	0	0	0	0	0	0	0	0	0	0	0	0	1	0	0	0	0	0	0	0	0	0	0	1	0	0	0	0	0	2
19. *Graphium pseudormiticum*	0	0	0	0	0	0	0	0	1	0	0	0	0	0	0	0	0	0	0	0	0	0	0	0	0	0	0	0	0	0	0	0	0	0	0	0	0	1
**Total**	**0**	**3**	**28**	**0**	**4**	**56**	**10**	**19﻿**	**18**	**2**	**0﻿**	**0**	**0**	**0﻿**	**34**	**2**	**2﻿**	**0**	**0**	**0﻿**	**12**	**21**	**28﻿**	**36**	**1**	**0﻿**	**0**	**9**	**0﻿**	**0**	**10**	**1**	**6**	**1**	**8**	**12**	**17**	**340**

^1^ Tree host species: I = *Pinus kesiya*; II = *P. armandii*; III = *P. yunnanensis*; IV = *P. semaonensis*; V = *Tsuga* sp. ^2^ Beetle species: A = *Coccotrypes cyperi*; B = *Cyrtogenius luteus*; C = *Hylurgops major*; D = *Ips acuminatus*; E = *Lasconotus* sp.; F = *Orthotomicus angulatus*; G = *Pissodes* sp.; H = *Polygraphus aterrimus*; I = *Polygraphus* sp.; J = *P. szemaoensis*; K = *P. verrucifrons*; L = *Stenoscelis* sp.; M = *Tomicus minor*; N = *T. piniperda*; O = *T. brevipilosus*; P = Unknown sp.1; Q = Unknown sp.2.* = information about specific niche not available.

Taxon 4 formed part of the *O. ips* complex (Fig. 1). This included 106 isolates (Table 3), nine of which were included in the analyses (Table 2). ITS and BT data (Suppl. material 2: Figure S4) confirmed that these isolates all represented *O. ips*.

**Figure 4.**
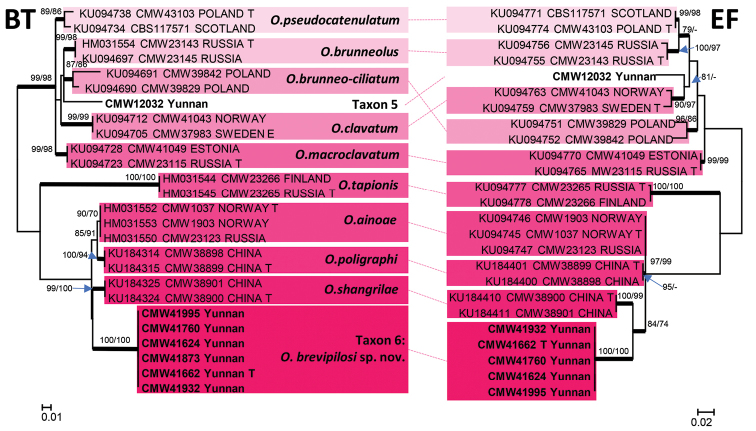
ML trees of the *O. clavatum* complex generated from DNA sequences of BT and EF regions. *Bold* branches indicate posterior probabilities values ≥ 0.95. Bootstrap values ≥ 70 % are recorded at nodes as ML/MP. T = ex-type isolates.

Taxon 5, including only one isolate, together with Taxon 6 that included eight isolates (Table 3), six for which sequences were produced, formed part of the *O. clavatum* complex (Fig. 1). BT and EF data confirmed that both these taxa grouped distinct from all known species in the complex (Fig. 4).

Five isolates comprised Taxon 7 (Tables 2 and 3), which formed part of the *O. piceae* complex (Fig. 1). Both BT and EF analyses confirmed that these isolates grouped with *O. setosum* isolates previously identified from Canada, Korea and China (Suppl. material 2: Figure S5).

There were 55 isolates (Table 3) belonging to the *O. ulmi* complex (Fig. 1). The sequences for these isolates were quite variable. Thus more detailed analyses, including all available related sequences from Genbank generated in previous studies dealing with the variable haplotypes of these taxa (Grobbelaar et al. 2009, Kamgan Nkuekam et al. 2010), were required. Taxon 8 represented *O. quercus* and Taxon 9 *O. tsotsi* based on ITS (Suppl. material 2: Figure S6), BT (Suppl. material 2: Figure S7), and EF (Suppl. material 2: Figure S8). Sequences of the 36 putative *O. quercus* isolates were very variable and respectively represented 13, 23 and 28 haplotypes for the ITS, BT and EF gene regions (Suppl. material 1: Table S2). When combined, 51 unique haplotypes were found (Suppl. material 1: Table S2). The 18 isolates representing Taxon 9 grouped with isolates of *O. tsotsi* (Suppl. material 2: Figures S6, S7, S8). The ITS data for *O. tsotsi* was less variable than those of *O. quercus* (Suppl. material 2: Figure S6), including only one haplotype. However, the BT (Suppl. material 2: Figure S7) and EF (Suppl. material 2: Figure S8) of *O. tsotsi* had 14 and 12 unique haplotypes respectively. The combined ITS, BT and EF data, represented 20 unique haplotypes (Suppl. material 1: Table S3). Based on BT (Suppl. material 2: Figure S7) and EF (Suppl. material 2: Figure S8) data, the isolate representing Taxon 10 grouped distinct from all other lineages and could represent an undescribed species.

**Figure 5.**
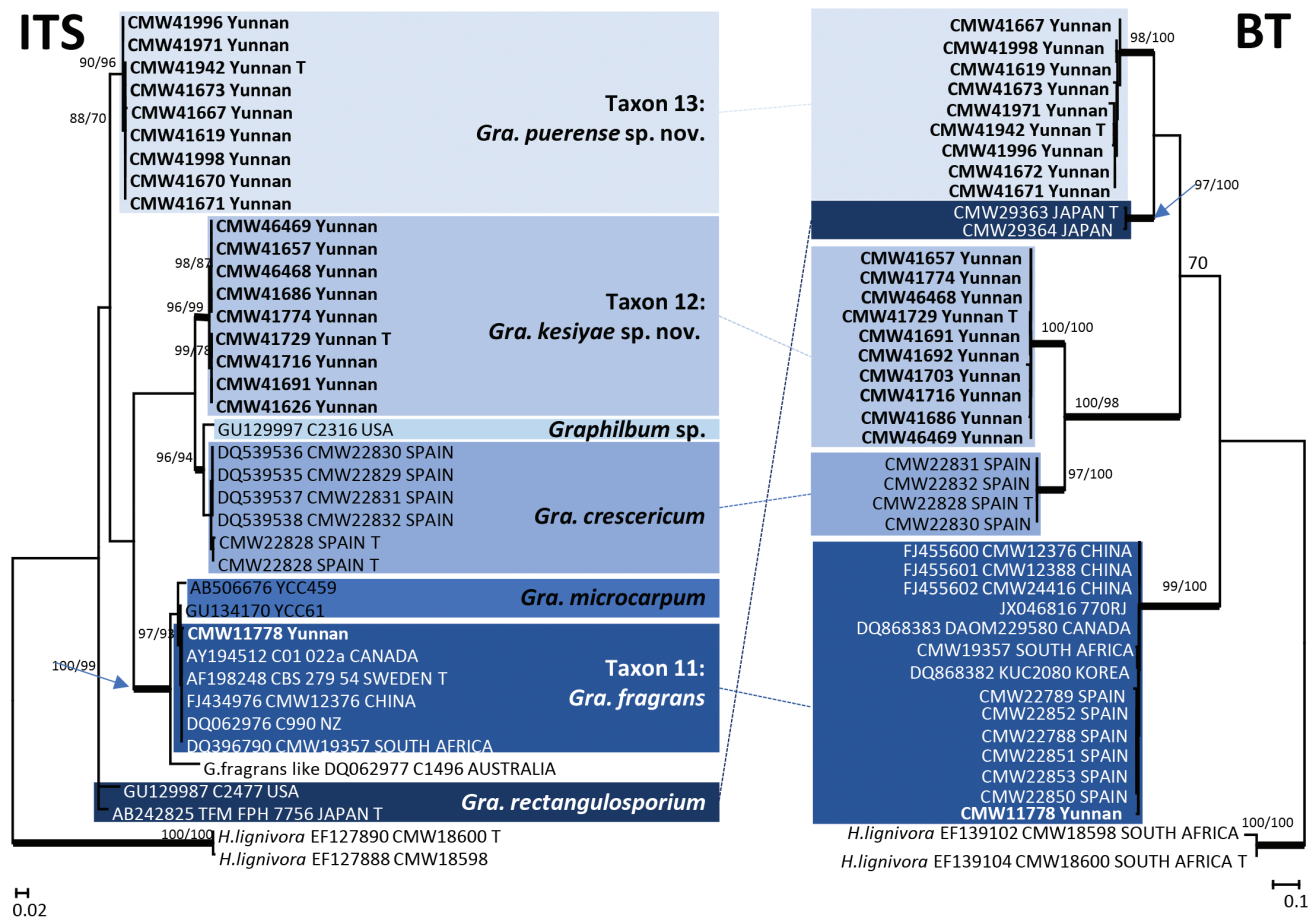
ML trees of the *Graphilbum* generated from DNA sequences of ITS and BT regions. *Bold* branches indicate posterior probabilities values ≥ 0.95. Bootstrap values ≥ 70 % are recorded at nodes as ML/MP. T = ex-type isolates.

For *Graphilbum*, results from the analyses of the ITS and BT sequences (Fig. 5) suggested that the Yunnan isolates resided in three taxa. These included one isolate of *Gra. fragrans* (Taxon 11) and two distinct, well-supported lineages (Taxa 12 and 13) each including 11 isolates (Table 3), representing undescribed species.

In *Leptographium* *s.l.* (Fig. 2), ten isolates (Taxon 14) grouped in the *L. procerum* complex (Table 3). Although both BT and EF sequences (Suppl. material 2: Figure S9) of this taxon differed in 1 bp from those of *L. gracile*, CAL sequences were identical to those of *L. gracile* and we conclude that Taxon 14 represents the latter species. However, species delineation in this complex requires reconsideration because sequences of several species in the complex are very similar.

A single isolate (Taxon 15) grouped in the *Grosmannia galeiformis* complex (Fig. 2, Table 2). Both the BT and EF sequences placed this isolate among those of *G. radiaticola* (Suppl. material 2: Figure S10).

**Figure 6.**
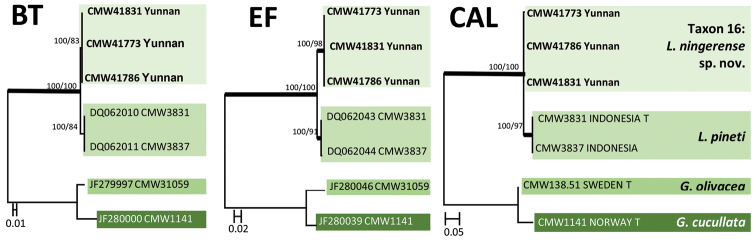
ML trees of the *L. pineti* generated from DNA sequences of BT, EF and CAL regions. *Bold* branches indicate posterior probabilities values ≥ 0.95. Bootstrap values ≥ 70 % are recorded at nodes as ML/MP. T = ex-type isolates.

Taxon 16 (Fig. 2) included 13 isolates (Table 3) that grouped closest to *L. pineti*, peripheral to the *G. olivacea* complex. BT, EF and CAL analyses (Fig. 6) showed that Taxon 16 was distinct from *L. pineti* and represented a novel species.

Forty one isolates (Tables 2 and 3) belonged to the *L. lundbergii* complex (Fig. 2). The analyses of BT and EF sequences (Suppl. material 2: Figure S11) showed that these isolates separated in two groups, respectively aligning with *G. yunnanensis* (Taxon 17) and *L. conjunctum* (Taxon 18).

A single isolate represented Taxon 19 (Table 2) in the genus of *Graphium*. Both the ITS and EF sequences of this isolate grouped with *Graphium pseudormiticum* isolates (Suppl. material 2: Figure S1).

### Frequencies of isolation

The origin and sources of the 340 isolates representing 19 taxa are presented in Tables 2, 3 and Suppl. material 1: S1. The 54 isolates collected from beetles and their galleries in 2001 and 2002 belonged to 11 taxa (Table 3). During the 2010 survey, 286 isolates belonging to 12 taxa were collected (Table 3). The 45 isolates collected directly from six bark beetle species belonged to seven taxa, the 184 isolates from mites to 11 taxa, and the 57 isolates from galleries represented nine taxa. Six taxa were present on beetles, galleries and mites, three taxa on galleries and mites, one taxon only on beetles, and two taxa only on mites. No taxa were found only in galleries that were not also found on beetles and/or mites. Four taxa were collected from both the 2001–2002 survey and the 2010 survey.

*Grosmannia yunnanensis*, *O. ips*, *O. quercus*, *S. nebularis* and Taxon 3 were the most frequently isolated species, representing 12.5%, 31.2%, 12.6%, 13.2% and 8.5% of the isolated fungi respectively. The remaining species were isolated only occasionally. *O. ips* was isolated from the niches (beetles, galleries, mites) of ten different bark beetles species. However, most of the *O. ips* were found associated with niches of *I. acuminatus*, *P. szemaoensis* and *C. cyperi* representing approximately 9.7%, 8.5% and 5.3%, respectively. *S. nebularis* was also isolated from the niches of ten beetle species and the highest frequency of isolation was 5.5%, associated with the niche of *O. angulatus*.

The number of fungal species isolated from different beetle or weevil niches varied between different species. There were ten fungal species, representing about 27.9% of total fungal isolates associated with the niche of *P. szemaoensis*. Among them, *O. ips* and *L. yunnanense* were the most frequently isolated, representing about 8.5% and 7.1%, respectively. There were eight fungal species, representing about 15.3%, associated with *Co. cyperi* and of these, the most frequently isolated fungus was *O. ips* with the frequency of 5.3%.

Only 13.2% of the total number of isolates were obtained directly from beetles (Table 3). Of these, 6.2% was obtained from *P. szemaoensis*. Most of the isolates from beetles were identified as *O. ips* (2.6%) and *O. quercus* (1.8%), while 2.9% and 2.6% of isolates were collected from *I. acuminatus* and *T. brevipilosus* respectively. Among these Taxon 6, representing 2.4%, was isolated only from *T. brevipilosus*. The other beetles vectored very low numbers of fungi. *O. ips* was the fungus most frequently isolated from the beetles, representing 4.4%, followed by Taxon 6 represting 2.4 % of the isolates.

Approximately 54% of all isolates were collected from mites (Table 3, Suppl. material 1: Table S1). The fungi associated with mites varied between the different mite species. Ten fungal species were isolated from *Insectolaelaps* sp. 1, representing 21.2% of the total. From some mite species, such as *Lasioseius* sp. 1, *Dendrolaelaps* sp. 2 and the unknown species in the families of Mesostigmata and Oribatei, only one fungal species was found, representing a frequency of isolation of 0.3%, 1.8%, 1.2% and 1.5%, respectively. *O. ips*, *S. nebularis* and *G. yunnanensis* were the three most frequently isolated species associated with mites, representing 15.3%, 10.6% and 7.9%, respectively.

## Taxonomy

Eight of the 19 taxa obtained in the present study represented undescribed species. For three of these, only a single isolate was obtained and we have chosen not to formally describe these. The remaining five taxa including two *Ophiostoma*, two *Graphilbum*, and one *Leptographium* species, are described as follows:

### Taxon 3

#### Ophiostoma acarorum

Taxon classificationFungiOphiostomatalesOphiostomataceae

R.Chang & Z.W.de Beer sp. nov.

823693

Fig. 7

##### Etymology.

The epithet *acarorum* refers to the subclass Acari in the Arachnida to which all mite species belong from which 25 of the 29 isolates of this species were isolated.

##### Description.

Sexual state not observed. Hyalorhinocladiella-like asexual state: *conidiophores* (7–) 18–76.5 (–140) μm long; *cells* arising directly from the hyphae, (10.5–) 13.5–24.5 (–31) × (1–) 1.5–2 (–2) μm; *conidia* hyaline, smooth, oblong, (3–) 3.5–5 (–6.5) × (0.7–) 1–1.5 (–2.5) μm.

**Figure 7.**
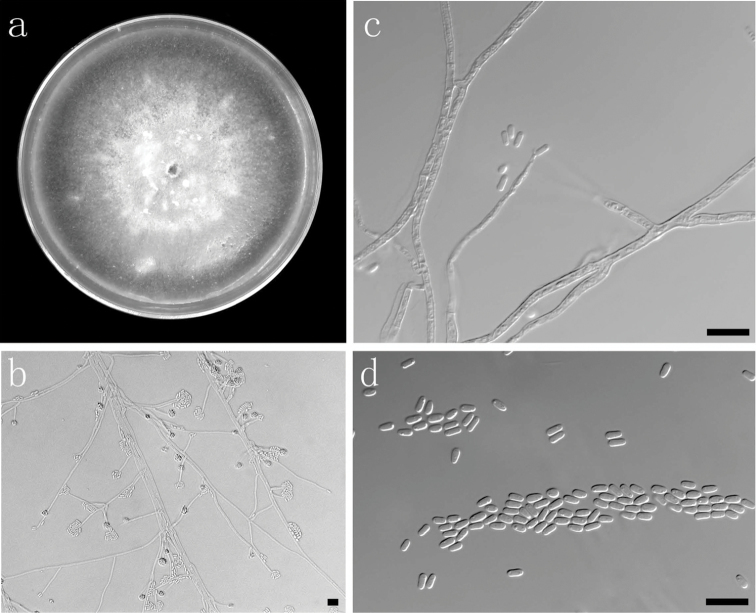
Morphological characters of asexual structures of *Ophiostoma acarorum* sp. nov. **a** fourteen days old culture on OA **b–c** Hyalorhinocladiella-like asexual state **d** conidia. Scale bars: **a–d** = 10 μm.

##### Culture characteristics.

Colonies hyaline at the beginning, becoming white to dark brown with age. Mycelium superficial on the 3% OA. Colony margin smooth. Colonies on 2% MEA flat, reaching 69 mm diam in 13 d at 30 °C. No growth observed at 5 °C. Optimal temperature for growth 25 °C.

##### Type material.

CHINA, Yunnan Province, Puer City, from *Insectolaelaps* sp. 1 in *Orthotomicus angulatus* gallery on *Pinus kesiya* bark, 17 Sep. 2010, *S.J.Taerum*, herbarium specimen of dried culture, PREM 61539 (holotype), CMW41850 = CBS139748 (ex-holotype culture).

##### Additional specimens examined.

CHINA, Yunnan Province, Puer City, from Histiostoma cf. sapromyzarum in *Cyrtogenius luteus* gallery on *Pinus kesiya* bark, 16 Sep. 2010, *S.J.Taerum*, PREM 61540, CMW41812 = CBS139658; from Histiostoma cf. sapromyzarum in *Cyrtogenius luteus* gallery on *Pinus kesiya* bark, 16 Sep. 2010, *S.J.Taerum*, CMW41798 = CBS139643.

##### Host.

*Pinus kesiya*.

##### Beetle vectors.

*Ips acuminatus*, *Polygraphus szemaoensis*.

##### Mite vectors.

Histiostoma cf. sapromyzarum (phoretic on *Cyrtogenius luteus*), *Insectolaelaps* sp. 1 (phoretic on *Ips acuminatus* and *Orthotomicus angulatus*).

##### Distribution.

At present known only from Yunnan, China.

##### Notes.

The hyalorhinocladiella-like asexual state of *O. acarorum* resembles that of *O. pallidulum* (Linnakoski et al. 2010), one of its two closest relatives based on phylogeny (Fig. 3). *Ophiostoma saponiodorum*, the other close relative has a similar hyalorhinocladiella-like state, but can be distinguished based on the presence of a second, synnematous asexual state (Linnakoski et al. 2010).

### Taxon 6

#### Ophiostoma brevipilosi

Taxon classificationFungiOphiostomatalesOphiostomataceae

R.Chang & Z.W.de Beer sp. nov.

823694

Fig. 8

##### Etymology.

The epithet *brevipilosi* refers to the bark beetle vector *Tomicus brevipilosus* from which all eight isolates of this taxon were obtained.

##### Description.

Sexual state not observed. Pesotum-like macronematal asexual state predominant. *Synnemata* simple, dark brown at the base, (179.5–) 227–468 (–667) μm long including *conidiogenous* *apparatus*, (22–) 32.5–58 (–69) μm wide at base; *cells* (13–) 16–26 (–32.5) μm long, *conidia* hyaline, 1-celled, smooth, oblong, (3–) 3–4.5 (–5.5) × (1.5–) 1.5–2.5 (–3) μm. Hyalorhinocladiella-like asexual state: *conidiophores* (14.5–) 33–115 (–145) μm long; *cells* arising directly from the hyphae, (12–) 15–38 (–47) × (1.1–) 1.5–2 (–2.5) μm; *conidia* hyaline, smooth, obovoid, (2.5–) 3–5.5 (–8) × (1.5–) 2–2.5 (–3) μm.

**Fig. 8.**
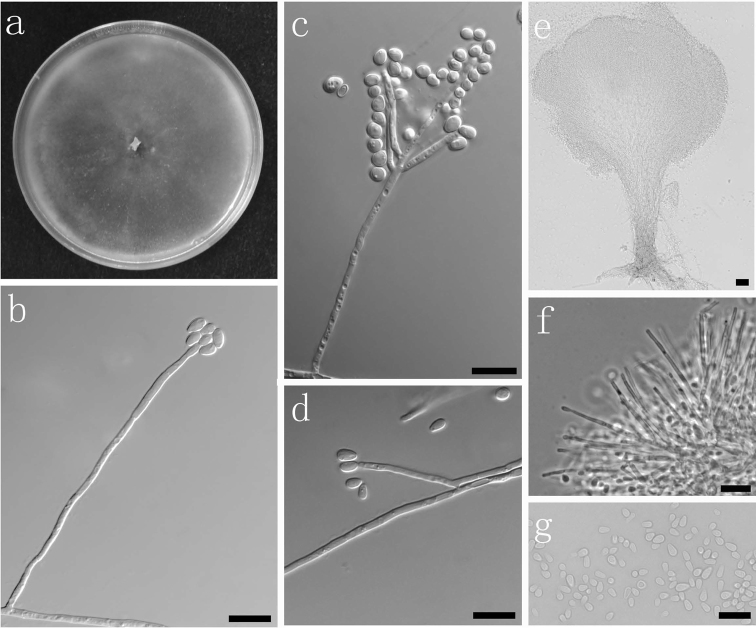
Morphological characters of asexual structures of *Ophiostoma brevipilosi* sp. nov. **a** fourteen days old culture on OA **b–d** Hyalorhinocladiella-like asexual state and condia **e** Pesotum-like macronematal asexual state **f** cells of Pesotum-like macronematal asexual state **g** conidia. Scale bars: **a–g** = 10 μm.

##### Culture characteristics.

Colonies hyaline at the beginning, then becoming white to dark. Mycelium superficial on the 3% OA. Colony margin smooth. Colonies on 2% MEA flat, reaching 67 mm diam in 11 d at 25 °C. No growth observed at 5 and above 30 °C. Optimal temperature for growth 20 and 25 °C.

##### Type material.

CHINA, Yunnan Province, Puer City, from *Tomicus brevipilosus* on *Pinus kesiya* bark, 27 Jun. 2010, *S.J.Taerum*, herbarium specimen of dried culture, PREM 61537 (holotype), CMW41873 = CBS139660 (ex-holotype culture).

##### Additional specimens examined.

CHINA, Yunnan Province, Puer City, from *Tomicus brevipilosus* on *Pinus kesiya* bark, 27 Jun. 2010, *S.J.Taerum*, PREM 61538, CMW41624 = CBS139661; CHINA, Yunnan Province, Puer City, from *Tomicus brevipilosus* on *Pinus kesiya* bark, 27 Jun. 2010, *S.J.Taerum*, CMW41662 = CBS139659.

##### Host.

*Pinus kesiya*.

##### Beetle vector.

*Tomicus brevipilosus*.

##### Distribution.

At present known only from Yunnan, China.

##### Notes.

The synnematous asexual state of *O. brevipilosi* corresponds with similar structures of *O. brunneo-ciliatum* as described by Linnakoski et al. (2016). The hyalorhinocladiella state resembles those of *O. brunneolum*, *O. macroclavatum*, *O. pseudocatenulatum* (Linnakoski et al. 2016) and *O. poligraphi* (Yin et al. 2016). However, the morphology of these structures is not sufficient to distinguish between the species in the complex, and DNA sequences of the BT and EF gene regions are recommended for accurate species identification.

### Taxon 12

#### Graphilbum kesiyae

Taxon classificationFungiOphiostomatalesOphiostomataceae

R.Chang & Z.W.de Beer sp. nov.

823695

Fig. 9

##### Etymology.

The epithet *kesiyae* refers to the tree host of all beetles and mites from which the 12 isolates of this species were collected.

##### Description.

Sexual state not observed. Pesotum-like macronematal asexual states predominant. *Synnemata* simple, dark brown at the base, (85.5–) 112.5–173 (–203) μm long including *conidiogenous apparatus*, (9–) 14–45.5 (–65.5) μm wide at base; *cells* (8.5–) 10–18.5 (–25.5) μm long; *conidia* hyaline, 1-celled, smooth, oblong, (3.5–) 4–5 (–5.5) × (1.5–) 1.5–2 (–2.5) μm. Hyalorhinocladiella-like asexual state: *conidiophores* (22–) 38–101.5 (–166) μm long; *cells* arising directly from the hyphae, (10–) 12–27(–40) × (1.2–) 1.5–2 (–2.5) μm; *conidia* hyaline, smooth, obovoid, (3.5–) 4–5.5 (–8.5) × (1–) 1.5–2 (–3) μm.

**Fig. 9.**
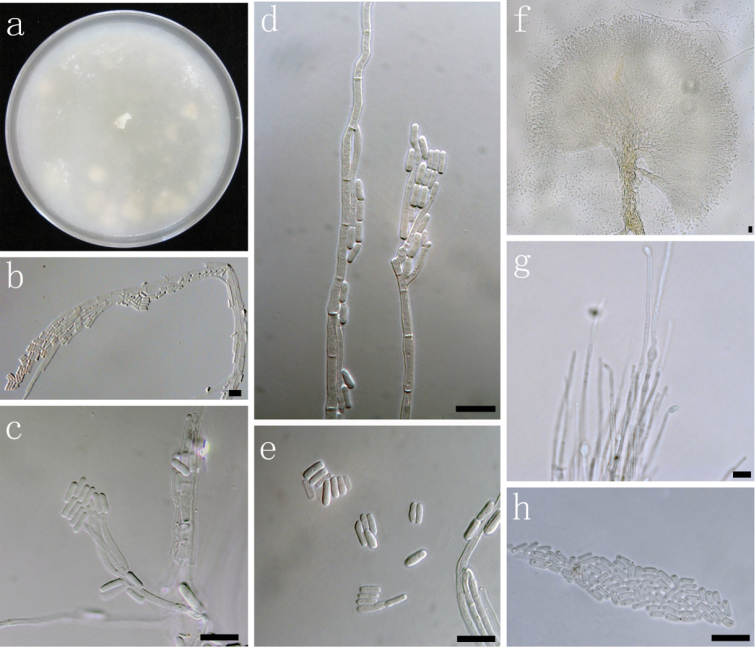
Morphological characters of asexual structures of *Graphilbum kesiyae* sp. nov. **a** fourteen days old culture on OA **b–e** Hyalorhinocladiella-like asexual state and conidia **f** Pesotum-like macronematal asexual state **g** cells of Pesotum-like macronematal asexual state **h** conidia. Scale bars: **a–h** = 10 μm.

##### Culture characteristics.

Colonies hyaline. Mycelium superficial on the 3% OA. Colony margin smooth. Colonies on 2% MEA flat, reaching 85 mm diam in 10 d at 25 °C. No growth observed at 5 and 35 °C. Optimal temperature for growth 25 °C.

##### Type material.

CHINA, Yunnan Province, Puer City, from *Polygraphus szemaoensis* gallery on *Pinus kesiya* bark, 12 Aug. 2010, *S.J.Taerum*, herbarium specimen of dried culture, PREM 61541 (holotype), CMW41729 = CBS139652 (ex-holotype culture).

##### Additional specimens examined.

CHINA, Yunnan Province, Puer City, from *Insectolaelaps* sp. 1 in *Polygraphus szemaoensis* gallery on *Pinus kesiya* bark, 10 Aug. 2010, *S.J.Taerum*, CMW41691 = CBS139642; CHINA, Yunnan Province, Puer City, from *Proctolaelaps nr. hystrix* in *Polygraphus szemaoensis* gallery on *Pinus kesiya* bark, 11 Aug. 2010, *S.J.Taerum*, PREM 61542, CMW41716 = CBS139657.

##### Host.

*Pinus kesiya*.

##### Beetle vectors.

*Polygraphus aterrimus, Polygraphus szemaoensis*.

**Mite vectors.** *Proctolaelaps nr. hystrix* (phoretic on *Polygraphus szemaoensis*), *Insectolaelaps* sp. 1 (phoretic on *Polygraphus szemaoensis*).

##### Distribution.

At present known only from Yunnan, China.

##### Notes.

*Graphilbum kesiyae* and *Gra. puerense* can be distinguished from *Gra. crescericum* by the presence of both synnematous and hyalorhinocladiella-like asexual states in culture. *Gra. crescericum* produces only the hyalorhinocladiella-like asexual state. The optimal temperature for growth of *Gra. puerense* is 30 °C while that for *Gra. kesiyae* is 25 °C, and synnemata of *Gra. puerense* reach double the length of those of *Gra. kesiyae*.

### Taxon 13

#### Graphilbum puerense

Taxon classificationFungiOphiostomatalesOphiostomataceae

R.Chang & Z.W.de Beer sp. nov.

823696

Fig. 10

##### Etymology.

The epithet *puerense* refers to the city from which this species was collected.

##### Description.

Sexual state not observed. Pesotum-like macronematal asexual states predominant. *Synnemata* simple, dark brown at the base, (187.5–) 206– 357(–437.5) μm long including *conidiogenous apparatus*, (12–) 15.5–45 (–61) μm wide at base; *conidiogenous* cells (15.5–) 18.5–30.5 (–34) μm long, *conidia* hyaline, 1-celled, smooth, oblong, (4–) 4–5 (–5.5) × (1–) 1.5–2 (–2.5) μm. Hyalorhinocladiella-like asexual state: *conidiophores* (17–) 3–140 (–232.5) μm long; *cells* arising directly from the hyphae, (6.5–) 10–25.5 (–42.5) × (1–) 1–2 (–3) μm; *conidia* hyaline, smooth, obovoid to oblong, (3.5–) 4–8 (–12) × (1–) 1.5–2.5 (–3) μm.

**Fig. 10.**
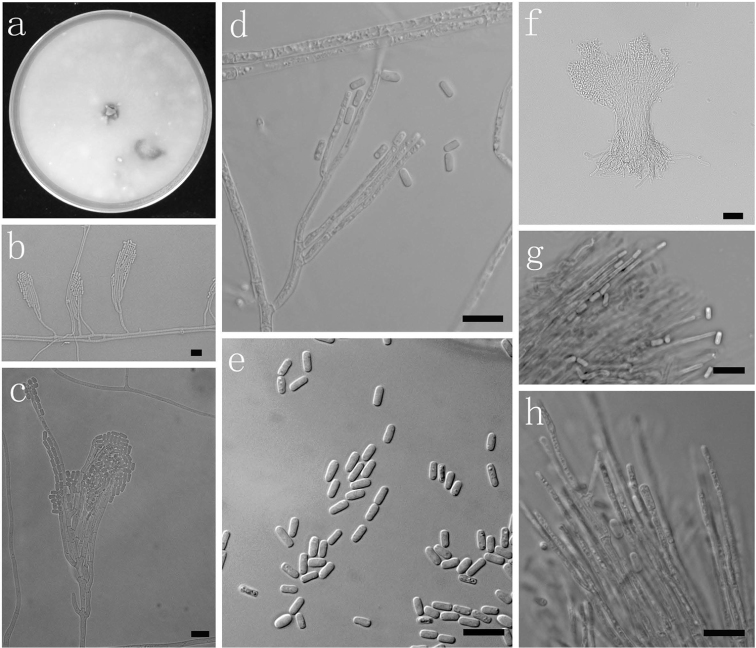
Morphological characters of asexual structures of *Graphilbum puerense* sp. nov. **a** fourteen days old culture on OA **b–d** Hyalorhinocladiella-like asexual state **e** conidia **f** Pesotum-like macronematal asexual state **g–h** cells of Pesotum-like macronematal asexual state and conidia. *Scale bars*: **a–h** = 10 μm.

##### Culture characteristics.

Colonies hyaline. Mycelium superficial on the 3% OA. Colony margin smooth. Colonies on 2% MEA flat, reaching 76 mm diam in 5 d at 30 °C. No growth observed at 5 °C. Optimal temperature for growth 30 °C.

##### Type material.

CHINA, Yunnan Province, Puer City, from *Polygraphus szemaoensis* gallery on *Pinus kesiya* bark, 29 Jun. 2010, *S.J.Taerum*, herbarium specimen of dried culture, PREM 61543 (holotype), CMW41673 = CBS139640 (ex-holotype culture).

##### Additional specimens examined.

CHINA, Yunnan Province, Puer City, from *Ips acuminatus* gallery on *Pinus kesiya* bark, 4 Jul. 2010, *S.J.Taerum*, PREM 61544, CMW41667 = CBS139651; CHINA, Yunnan Province, Puer City, from *Ips acuminatus* gallery on *Pinus kesiya* bark, Jul 2010, *S.J.Taerum*, CMW41942 = CBS139650.

##### Host.

*Pinus kesiya*.

##### Beetle vectors.

*Ips acuminatus*, *Polygraphus szemaoensis*.

##### Mite vectors.

*Proctolaelaps nr. hystrix* (phoretic on *Ips acuminatus*), *Insectolaelaps* sp. 1 (phoretic on *Ips acuminatus*), and *Uropodoidea* sp. 2 (phoretic on *Polygraphus szemaoensis*).

##### Distribution.

At present known only from Yunnan, China.

##### Notes.

See comparison between *Gra. kesiyae* and *Gra. puerense* above under notes of *Gra. kesiyae*.

### Taxon 16

#### Leptographium ningerense

Taxon classificationFungiOphiostomatalesOphiostomataceae

R.Chang & Z.W.de Beer sp. nov.

823697

Fig. 11

##### Etymology.

The epithet *ningerense* refers to the Ning’er county where all isolates of this taxon were collected.

##### Description.

Sexual state not observed. Asexual state, *conidiophores* occurring singly or in groups of up to 3, macronematous, mononematous, erect, arising directly from the mycelium, (93.5–) 141.5–195.5 (–210.5) μm long. Rhizoids present. *Stipes* dark olivaceous, 4–6 septa, not constricted at septa, (66–) 119.5–142 (–159) μm long. *Apical* cells not swollen at apex, (3–) 5–6.5 (–7) μm wide. *Basal cells* occasionally swollen at apex, (5.5–) 7–10 (–11.5) μm wide. *Conidiogenous apparatus* (28–) 35–58 (–70) μm long, excluding the conidial mass, with multiple series of cylindrical branches. *Primary branches* olivaceous, smooth, cylindrical, not swollen at apex, aseptate, arrangement of primary branches was Type B—two or more branches, (12.5–) 14.5–18 (–19.5) × (3.5–) 4–5.5(–6.5) μm. *Secondary branches* light olivaceous, frequently swollen at apex, aseptate, (6.5–) 9–13(–15) × (3.5–) 4–5 (–5.5) μm. *Tertiary branches* light olivaceous, aseptate, (7–) 8–10 (–12) × (3–) 3.5–4.5 (–5) μm. *Conidiogenous cells* discrete, hyaline, 2–3 per branch, aseptate, cylindrical, tapering slightly at the apex, (10.5–) 12–17.5 (–20.5) × (2–) 2–2.5 (–3) μm. *Conidia* hyaline, aseptate, elliptical, (3–) 3.5–5.5 (–6.5) × (1.5–) 2–3 (–4) μm.

**Fig. 11.**
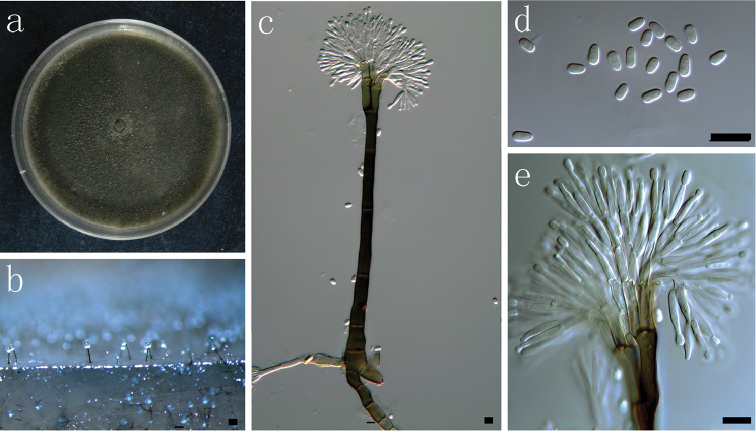
Morphological characters of asexual structures of *Leptographium ningerense* sp. nov. **a** fourteen days old culture on OA **b** mononematous asexual morph on wood tissue on WA **c** conidiophore **d** conidia **e** conidiogenous apparatus. Scale bars: **a–e** = 10 μm.

##### Culture characteristics.

Colonies on 3% OA flat, hyaline at the beginning, then becoming light olivaceous to dark olivaceous. Colonies hyaline at the beginning, then becoming dark olivaceous. Mycelium superficial on the 3% OA with olivaceous aerial mycelium. Colony margin smooth. Conidiophores forms abundantly in clusters on OA. Colonies on 2% MEA flat, reaching 76 mm diam in 10 d at 25 °C. No growth observed at 5 and 35 °C. Optimal temperature for growth 25 °C, reaching 30.5 mm in diam. in 7 days.

##### Type material.

CHINA, Yunnan Province, Puer City, from Schwiebea (Jacotietta) taiwanensis hyperphoretic on *Coccotrypes cyperi* on *Pinus kesiya* bark, 16 Sep. 2010, *S.J.Taerum*, herbarium specimen of dried culture, PREM 61545 (holotype), CMW41786 = CBS139663 (ex-holotype culture).

##### Additional specimens examined.

CHINA, Yunnan province, Puer City, from *Insectolaelaps* sp. 1 in *Orthotomicus angulatus* on *Pinus kesiya* bark, 17 Sep. 2010, *S.J.Taerum*, PREM 61546, CMW41831 = CBS139664.

##### Host.

*Pinus kesiya*.

##### Beetle vectors.

*Polygraphus szemaoensis*.

##### Mite vectors.

*Dendrolaelaps* sp. 1 (phoretic on *Coccotrypes cyperi*), *Dendrolaelaps* sp. 2 (phoretic on *Coccotrypes cyperi* and *Cyrtogenius luteus*), Schwiebea (Jacotietta) taiwanensis (phoretic on *Coccotrypes cyperi*), *Insectolaelaps* sp. 1 (*Orthotomicus angulatus*).

##### Distribution.

At present known only from Yunnan, China.

##### Notes.

*L. ningerense* is morphologically similar to *L. pineti*, but grows much more rapidly, reaching 30 mm in 7 d on 2% MEA at 25 °C while *L. pineti* reaches a diameter of only 15 mm in 6 d. However, the two species are best distinguished with BT, EF and CAL sequences.

## Discussion

This study resulted in a total of 340 fungal isolates of ophiostomatoid fungi obtained from the beetles and weevils, their galleries and phoretic mites in a province of China where these fungi are poorly known. The fungi resided in the two phylogenetically unrelated Microascales and Ophiostomatales and included a species of *Graphium* (Microascales) as well as species of *Sporothrix*, *Graphilbum*, *Leptographium* and *Ophiostoma* (Ophiostomatales). Analysis showed that these isolates belonged to 19 distinct taxa, eight of which represented undescribed species of which five were provided with names. Of the remaining 11 species, 10 had previously been reported from China, with only *S. nebularis* representing a new report for this country. This study also includes the first records of fungi associated with the Scolytine beetle species *Coccotrypes cyperi, Cyrtogenius luteus*, and *Tomicus brevilopus* and it is the first time that fungal associates are reported from beetle-associated mites in China.

Surveys in this study aimed to explore fungal diversity in conifer-infesting beetle ecosystems in Yunnan. This is in contrast to determining the specificity of these interactions where a much more focused and systematic sampling would have been required. The exact nature of the interactions between the beetles and mites, mites and fungi, beetles and fungi, and all of these with their host trees, thus remains largely unknown. The discussion below therefore focuses on the fungi collected in these surveys, with limited notes on the hosts, beetles and mites and some general observations.

The two *Sporothrix* species collected in this study were primarily from mites. *Sporothrix nebularis* was the second most abundant species and 36 of the 45 isolates came from five different mite species phoretic on five different beetle species. The fungus was first described from *Hylastes attenuatus* infesting *Pinus radiata* in Spain (Romón et al. 2014b) and was later also found on *Hylastes ater* and *Hylurgops palliatus* from the same host and country (Romón et al. 2014a). Our results suggest that *S. nebularis* has a much wider Eurasian distribution and host range than previously realized. It is also possible that in Europe, many of the previous reports of *S. stenoceras* from nine bark beetle species (Kirisits 2004), actually represent *S. nebularis*. This is particularly because the two taxa are morphologically almost indistinguishable, and *S. nebularis* might have been misidentified as *S. stenoceras* where only morphology was considered (Romón et al. 2014b). The single isolate of *Sporothrix* sp. A was from a mite on *Cyrtogenius luteus*. Overall, the results support the suggestion by De Beer et al. (2016a) that the majority of *Sporothrix* species are mite associates.

*Ophiostoma acarorum* grouped peripheral to the well-known species complexes in *Ophiostoma* *s.str.*, and closest to, but distinct from *O. pallidulum* and *O. saponiodorum. O. pallidulum* is known from ten different bark beetle and one weevil species infesting *Pinus sylvestris* in Finland (Linnakoski et al. 2010), Poland (Jankowiak and Bilański 2013a, b, c), and the Ukraine (Davydenko et al. 2017). Interestingly, *O. pallidulum* was also isolated from dead *P. sylvestris* roots in Poland in the absence of beetle attack (Jankowiak et al. 2012). *O. saponiodorum* is known from two spruce-infesting beetles in Finland and Russia (Linnakoski et al. 2010), pine-infesting *Hylastes* and *Pissodes* spp. in Poland and Spain (Jankowiak and Bilański 2013a, c, Romón et al. 2014a), and *Orthotomicus erosus* caught in traps in Italy (Malacrinò et al. 2017). Similar to *O. pallidulum* and *O. saponiodorum*, *O. acarorum* does not appear to be beetle-specific as it was isolated from three mite species phoretic on four beetle species, and from the galleries of two beetle species. However, the fact that 25 of 29 isolates in the present study were from mites, suggests that *O. acarorum* is a symbiont of mites on conifer-infesting beetles, rather than the beetles themselves. The wide range of beetle species from which *O. pallidulum* and *O. saponiodorum* have been isolated in Europe, always in relatively low abundance, also suggests that these two species are mite rather than beetle symbionts.

*Ophiostoma ips* was the species most frequently isolated in the study, constituting 31% of the 340 isolates. It was also the fungus found in association with the largest number of beetle species i.e. 10 of 17, and with three of the five host trees in central and southern Yunnan. This is consistent with current knowledge of the fungus that is known to have a global distribution (Zhou et al. 2007) and is associated with a wide range of conifer-infesting bark beetles in various genera (Kirisits 2004, Rumbold 1931, Upadhyay 1981). Shortly after the description of *O. ips* (Rumbold 1931), Leach et al. (1934) had already shown that ascospores of the fungus were vectored by mites associated with two *Ips* spp. Furthermore, Stone and Simpson (1990) observed that mycetophagous mites associated with *Ips grandicollis* fed directly on *O. ips* in the galleries of the beetle. About half of the 106 *O. ips* isolates from Yunnan came from mites associated with six of the bark beetle species. The results thus provide additional weight to the hypothesis that *O. ips* is primarily a mite associate, rather than a bark beetle associate.

Two taxa from Yunnan formed part of the *O. clavatum* complex as recently defined by Linnakoski et al. (2016). These authors showed that most of the species in the complex appear to be specific to one or two closely related *Ips* spp. on either a pine or spruce host from Europe. The only exception is *O. macroclavatum*, that is known from on three *Ips* spp. and *Pityogenes chalcographus* on both pine and spruce. Yin et al. (2016) subsequently described two additional species in the complex from *Polygraphus poligraphus* and *Ips shangrila* respectively, on spruce in Qinghai Province. Both fungi were also isolated from *Dendroctonus micans* in the same area. One of the species that we collected, *O. brevipilosi*, was from *Tomicus brevipilosus* beetles infesting *P. kesiya*. It is thus the first species in the complex associated with a *Tomicus* species. The other species from Yunnan, *Ophiostoma* sp. B (Taxon 5) was represented by only one isolate from the gallery of an unknown beetle on *P. semaonensis*.

Only a single species of the *O. piceae* complex, *O. setosum*, was obtained during the surveys. Five isolates of this species were from *Pissodes* galleries on *Tsuga dumosa*, confirming a previous report from the same host in China (Paciura et al. 2010b). The fungus was originally described from *Tsuga heterophylla* in Canada (Uzunovic et al. 2000). It was also reported from *Tsuga* and *Pseudotsuga* in the USA (Harrington et al. 2001), from *Hylastes ater* on pine in New Zealand (Reay et al. 2005) and pine logs imported from New Zealand to Korea (Kim et al. 2005a). In Bhutan, this species is associated with *Ips schmutzenhoferi* on *Picea spinulosa* and *Pinus wallichiana* (Kirisits et al. 2012). It thus seems as if *O. setosum* is a generalist associate of beetles infesting conifers in native Northern Hemisphere forests, and was most likely introduced into New Zealand.

The third most abundant species collected in this study was *O. quercus* that forms part of the *O. ulmi* species complex (De Beer and Wingfield 2013). This fungus is generally considered as hardwood-infecting with many reports from wounds and stained wood of well-known tree genera such as *Quercus*, *Betula*, *Populus*, *Eucalyptus*, *Acacia*, and *Nothofagus* from North and South America, Europe, Africa, Australasia, and Central and East Asia (De Beer et al. 2003, De Errasti et al. 2016, Grobbelaar et al. 2009, Harrington et al. 2001, Paciura et al. 2010b). Little is known regarding the insect vectors of *O. quercus* apart from reports that the fungus is vectored by nitidulid beetles on oak (Juzwik et al. 1998) and eucalypts (Kamgan Nkuekam et al. 2012). Unlike most other species in the *O. ulmi* complex, there is a growing body of evidence suggesting that *O. quercus* is also a successful colonist of conifer wood. It has been reported from stained pine wood in the USA, Sweden, Australia, New Zealand and South Africa (De Beer et al. 2003, Harrington et al. 2001), but also in association with pine bark beetles such as *Tomicus yunnanensis* in China (Paciura et al. 2010b), *Hylastes plumbeus* in Japan (Masuya et al. 2009), as well as *Ips sexdentatus* and *Hylobius abietus* in Poland (Jankowiak 2012, Jankowiak and Bilański 2013b). Furthermore, it has been found with *Pissodes* weevils on *Tsuga* in China (Paciura et al. 2010b) and pine in Poland (Jankowiak and Bilański 2013c).

The 43 *O. quercus* isolates obtained in this study came from four of the host trees distributed in eight of the ten study sites in both central and southern Yunnan. The *O. quercus* isolates were found in association with eight beetle species, with 13 of the isolates coming from three mite species. To the best of our knowledge, this represents the first records of this fungus from mites. However, spores of the closely related and morphologically similar *O. novo-ulmi* have been observed in the guts and sporothecae of mites phoretic on *Scolytus* beetle vectors of the Dutch Elm Disease fungi (Brasier 1978, Moser et al. 2010). The difference between the latter group of fungi and *O. quercus* is that they are specific to elm trees and elm-infesting beetles. In contrast, *O. quercus* is probably of all ophiostomatoid species the one with the widest array of hosts and the plasticity to utilize as a vector, any arthropod passing a wound or gallery in which it is growing. It is thus not surprizing that *O. quercus* is also one of the most genetically diverse ophiostomatoid species (Grobbelaar et al. 2009, Kamgan Nkuekam et al. 2010), which is supported by the large variety of haplotypes revealed in analyses of the BT and EF gene regions in this study.

*Ophiostoma tsotsi* was the second species in the *O. ulmi* complex obtained in this study. This species, which is morphologically indistinguishable from *O. quercus*, is known from wounds on planted *Eucalyptus* and *Acacia mearnsii* trees in Africa (Grobbelaar et al. 2010), Australia (Kamgan Nkuekam et al. 2011) and China (Grobbelaar et al. 2011), as well as native mangroves in South Africa (Osorio et al. 2016). The only insects from which the fungus has previously been reported are nitidulid beetles (Kamgan Nkuekam et al. 2012). We isolated *O. tsotsi* from galleries of two pine bark beetles, as well as from two mite species associated with one of these beetles. This was an unexpected result because, much like *O. quercus*, the assumption has been that *O. tsotsi* is an exclusively hardwood-infesting member of the *O. ulmi* complex. This complex was initially referred to as the ‘hardwood lineage’ of the *O. piceae* complex (De Beer et al. 2003, Harrington et al. 2001) before De Beer and Wingfield (2013) distinguished between the *O. piceae* and *O. ulmi* complexes. Although *O. tsotsi* is known from fewer hosts and locations than *O. quercus*, it also displays a relatively high level of genetic diversity as was evident from our BT and EF sequence analyses.

A single isolate referred to as *Ophiostoma* sp. C (Taxon 10) came from an unknown beetle gallery on *Tsuga*. This isolate was clearly distinct from all other species in the *O. ulmi* complex. Although we have chosen not to describe it as new, together with *O. quercus* and *O. tsotsi*, this is only the third of the 18 species currently recognized in the complex to be reported from conifer hosts.

Three of the taxa collected in the surveys resided in the genus *Graphilbum* that includes 10 known species and several undescribed taxa (De Beer and Wingfield 2013, Lu et al. 2009a, Reid and Hausner 2015, Romón et al. 2014b). All the known species are from conifers (*Abies*, *Picea*, *Pinus*, *Tsuga*) and mostly from bark beetles or their galleries, and in some cases stained wood. Only two undescribed species are from hardwoods (Geldenhuis et al. 2004, Kamgan Nkuekam et al. 2008). *Graphilbum fragrans* is by far the most common species with one report from bark beetles on spruce and fir in Canada (Jacobs et al. 2003). The remainder of the species have been collected from a variety of pine bark beetles and weevils from Europe (Mathiesen-Käärik 1953, Romón et al. 2007), the USA, New Zealand and Australia (Harrington et al. 2001), South Africa (Zhou et al. 2006), Japan (Masuya et al. 2009) and Korea (Kim et al. 2007).

*Graphilbum fragrans* found in this study had previously been reported from *T. yunnanensis* on *Pinus yunnanensis*, and *Pissodes* spp. on *Tsuga dumosa* and *P. armandii* in China (Paciura et al. 2010b, Zhou et al. 2013). In the present study, the fungus was isolated from galleries of *Tomicus minor* on *P. kesiya*. We collected 11 isolates each for *Gra. kesiyae* (Taxon 12) and *Gra. puerense* (Taxon 13). *Gra. kesiyae* was found in association with three beetle species with seven isolates from mites. *Gra. puerense* was from two beetle species with three isolates from mites. These are the first reports of any *Graphilbum* species isolated from mites.

Five species of fungi residing in *Leptographium* *sensu lato* were collected in this study, including one novel species. Ten isolates representing Taxon 14 were from *Pissodes* sp. on *P. armandii* and were shown to represent *L. gracile*. This species was described from the same insects and tree host as the isolates in the present study, as well as from *Ips typographus* on *Picea koraiensis* (Paciura et al. 2010a). Four other species grouped very closely to *L. gracile* in the *L. procerum* complex. *Leptographium sinoprocerum* and *L. sinense* were both described from China where they were associated with the introduced bark beetle *Dendroctonus valens* that infests and kills native *P. tabuliformis* (Lu et al. 2008), and native *Hylobitelus xiaoi* on non-native *P. elliottii* (Yin et al. 2015). *Leptographium longiconidiophorum* was originally isolated from *P. densiflora* in Japan (Yin et al. 2015) and *L. bhutanense* from *Hylobitelus chenkupdorjii* on *Pinus wallichiana* in Bhutan (Zhou et al. 2008).

A single isolate from the gallery of an unknown bark beetle on *Tsuga* grouped with *Grosmannia radiaticola*. This species was originally described from stained *Pinus radiata* wood imported from New Zealand to Korea (Kim et al. 2005b). However, it has been reported from a variety of pine root-infesting bark beetles from South Africa, Chile, Sweden (Linnakoski et al. 2012), California (Kim et al. 2011) and Poland (Jankowiak and Bilański 2013a, b). In China, it has been reported from a gallery of the invasive *D. valens* on *P. tabuliformis* in Shanxi, which might have resulted in the in the impression that the fungus was introduced with the beetle into China (Lu et al. 2009b). However, the present report from another conifer host in a distant province suggests that the fungus could be native across Eurasia.

Of the 13 isolates of *L. ningerense* collected in this study one isolate was from the gallery of *Polygraphus szemaoensis* on *Pinus kesiya*, while the remaining 12 isolates were from four mite species associated with *Coccotrypes cyperi*, *Cyrtogenius luteus* and *Orthotomicus angulatus*. This species grouped closest to *L. pineti*, which was described from the gallery of an *Ips* sp. under the bark of *Pinus merkusii* growing on the island of Sumatra, Indonesia (Jacobs et al. 2000). The fact that no isolates were obtained from beetles might indicate that this species is a preferential symbiont of mites.

The fourth most prevalent species collected in this study was *G. yunnanensis* including 39 isolates, 27 of which were vectored by seven mite species. *G. yunnanensis* was originally described from *Tomicus yunnanensis* on *P. yunnanensis* in China (Zhou et al. 2000), and was subsequently reported from *Polygraphus major* on *Pinus kesiya* in Thailand (Yamaoka et al. 2007), and four other pine bark beetle species on *P. densiflora* in Japan (Masuya et al. 2009). Results of the present study have added an additional three beetle species to this list including *Cryphales luteus*, *Polygraphus szemaoensis* and an unidentified *Polygraphus* sp. This seemingly promiscuous relationship of *G. yunnanensis* with nine different beetle species in East Asia, together with the fact that more than two thirds of the isolates in our study were from mites, suggests an association of this fungus with mites, rather than beetles.

Two isolates of *L. conjunctum*, including one from a mite, were collected in the present study. *L. conjunctum* has not previously been reported from any location other than in the case of the original study in which it was described from *Hylurgops major* on *Pinus yunnanensis* in China (Paciura et al. 2010a). Our one isolate was from *H. major*, while the mite from which the second isolate came, was collected from the unknown *Polygraphus* sp.

The single *Graphium* isolate arising from this study was from a mite on *Ips acuminatus*, and had identical sequences to *Gr. pseudormiticum*. This fungus was first described from South Africa in association with *Orthotomicus erosus* on pine bait logs (Mouton et al. 1994). It was subsequently found in Sweden associated with *Ips typographus* on an unknown host tree, in Austria with *Tomicus minor* on *Pinus sylvestris* (Lackner and de Hoog 2011), and in China associated with *Pissodes* sp. on *Tsuga dumosa* (Paciura et al. 2010b). This is the first report of a *Graphium* species associated with a mite. The low frequency with which it was encountered could result from the fact that *Graphium* species are unable to grow on media including the antibiotic cycloheximide, which is commonly used as in a selective medium for the Ophiostomatales in surveys of bark beetle fungi.

## Conclusions

Three surveys of pine-infesting bark beetles in Yunnan revealed several new fungal species and new beetle-fungus associations. This supports the view that the diversity of fungi associated with bark beetles in China is high and that it deserves further exploration. The results of the 2010 survey that included isolations from mites, revealed that many of the ophiostomatoid species often considered as beetle associates can also be isolated frequently from mites. It has been suggested that mycetophagous mites are often generalists with the ability to feed on and vector several fungal species (Hofstetter et al. 2013). It is also known that phoretic mites are promiscuous in terms of their beetle vectors, and that they will infest virtually any beetle species in order to reach their tree hosts (Hofstetter et al. 2013). Our results underscore these views although structured surveys supported by statistical analyses should be undertaken to better understand these relationships.

One of the most surprising and intriguing outcomes of the study was that fungal species such as *O. quercus* and *O. tsotsi*, which were considered to be primarily hardwood-infecting species, are prevalent on several of the pine-specific beetles and their associated mites. Bark beetles commonly have broad host ranges on either the hardwoods or conifers, but not across these groups. The question thus arises how these hardwood-infecting fungi can move between these host groups. One possibility is that they simply move with mites from one tree in a forest to another, crossing the forest floor and carrying the fungi with them. However, it is also known that most bark beetle-associated mites are not monospecific and may be common on other non-bark beetle hosts as well (e.g. Tenebrionidae, Cleridae, Histeridae, Elateridae) (Hofstetter et al. 2013). Some of these often predatory beetles, could thus vector the mites and their fungi across the otherwise hardwood-conifer barrier. The determining factor would then be whether the fungus has the ability to survive in the new environment. The results of this study suggest that *O. quercus* and *O. tsotsi* are the rare exceptions among the ophiostomatoid fungi that are equally fit in both the angiosperm and gymnosperm environments.

None of the known fungal species collected in the present study are considered pathogens based on current published knowledge, and we have not found any evidence that any of the novel taxa are pathogens. However, the study underscores the possibility that many ophiostomatoid fungi that have been considered beetle associates in the past, might actually have a closer association with mites, or that mites at least have the ability to vector them. Generally, bark beetles and their fungal associates are considered serious threats with invasive potential when they are introduced into new environments through the movement of wood and wood products (Wingfield et al. 2017). However, most pest risk analyses consider only the beetles and the fungi. Our results suggest that even in cases where beetles do not successfully establish and reproduce in a new environment, mites could vector potential fungal pathogens and introduce them into established beetle ecosystems. The role of mites in mixing fungal assemblages of different beetle species on a single tree or log, especially in the case of invasive versus native beetle species, deservers futher exploration.

## Supplementary Material

XML Treatment for Ophiostoma acarorum

XML Treatment for Ophiostoma brevipilosi

XML Treatment for Graphilbum kesiyae

XML Treatment for Graphilbum puerense

XML Treatment for Leptographium ningerense

## Supplementary materials

Supplementary material 1 Table S1–S3 Data type: species data Explanation note: Table S1. Numbers of ophiostomatoid fungal isolates obtained from different mite species in this study. Table S2. Haplotypes of *Ophiostoma quercus*. Table S3. Haplotypes of *Ophiostoma tsotsi*. This is an open access article distributed under the terms of the Creative Commons Attribution License (CC BY 4.0), which permits unrestricted use, distribution, and reproduction in any medium, provided the original author and source are credited.Runlei Chang, Tuan A. Duong, Stephen J. Taerum, Michael J. Wingfield, Xudong Zhou, Z. Wilhelm de Beer

Supplementary material 1 Figure S1–S11 Data type: occurence Explanation note: Figure S1. Map of Yunnan Province in China showing the sites and tree hosts from which samples were collected during the three surveys in 2001, 2002 and 2010. Figure S2. ML trees of the genus *Graphium* generated from DNA sequences of the ITS and BT regions. *Bold* branches indicate posterior probabilities values ≥ 0.95. Bootstrap values ≥ 70 % are recorded at nodes as ML/MP. T = ex-type isolates. Figure S3. ML trees of the genus *Sporothrix* generated from DNA sequences of ITS and BT regions. *Bold* branches indicate posterior probabilities values ≥ 0.95. Bootstrap values ≥ 70 % are recorded at nodes as ML/MP. T = ex-type isolates. Figure S4. ML trees of the *O. ips* complex generated from DNA sequences of ITS and BT regions. *Bold* branches indicate posterior probabilities values ≥ 0.95. Bootstrap values ≥ 70 % are recorded at nodes as ML/MP. T = ex-type isolates. Figure S5. ML trees of the *O. piceae* complex generated from DNA sequences of BT and EF regions. *Bold* branches indicate posterior probabilities values ≥ 0.95. Bootstrap values ≥ 70 % are recorded at nodes as ML/MP. T = ex-type isolates. Figure S6. ML tree of the *O. quercus* complex generated from DNA sequences of ITS region. *Bold* branches indicate posterior probabilities values ≥ 0.95. Bootstrap values ≥ 70 % are recorded at nodes as ML/MP. T = ex-type isolates. Figure S7. ML tree of the *O. quercus* complex generated from DNA sequences of BT region. *Bold* branches indicate posterior probabilities values ≥ 0.95. Bootstrap values ≥ 70 % are recorded at nodes as ML/MP. T = ex-type isolates. Figure S8. ML tree of the *O. quercus* complex generated from DNA sequences of EF region. *Bold* branches indicate posterior probabilities values ≥ 0.95. Bootstrap values ≥ 70 % are recorded at nodes as ML/MP. T = ex-type isolates. Figure S9. ML trees of the *L. procerum* complex generated from DNA sequences of BT, EF and CAL regions. *Bold* branches indicate posterior probabilities values ≥ 0.95. Bootstrap values ≥ 70 % are recorded at nodes as ML/MP. T = ex-type isolates. Figure S10. ML trees of the *G. galeiformis* complex generated from DNA sequences of BT and EF regions. *Bold* branches indicate posterior probabilities values ≥ 0.95. Bootstrap values ≥ 70 % are recorded at nodes as ML/MP. T = ex-type isolates. Figure S11. ML trees of the *L. lundbergii* complex generated from DNA sequences of BT and EF regions. *Bold* branches indicate posterior probabilities values ≥ 0.95. Bootstrap values ≥ 70 % are recorded at nodes as ML/MP. T = ex-type isolates. This is an open access article distributed under the terms of the Creative Commons Attribution License (CC BY 4.0), which permits unrestricted use, distribution, and reproduction in any medium, provided the original author and source are credited.Runlei Chang, Tuan A. Duong, Stephen J. Taerum, Michael J. Wingfield, Xudong Zhou, Z. Wilhelm de Beer
