# Supplementary material for: Ophiostomatoid fungi associated with conifer-infesting beetles and their phoretic mites in Yunnan, China
Source: MycoKeys. 2017 Dec 21;(28):19–64. doi: 10.3897/mycokeys.28.21758 (PMC5804140; doi:10.3897/mycokeys.28.21758)
Supplement: Supplementary material 1 — Table S1–S3 [file mycokeys-28-019-s003.pdf]

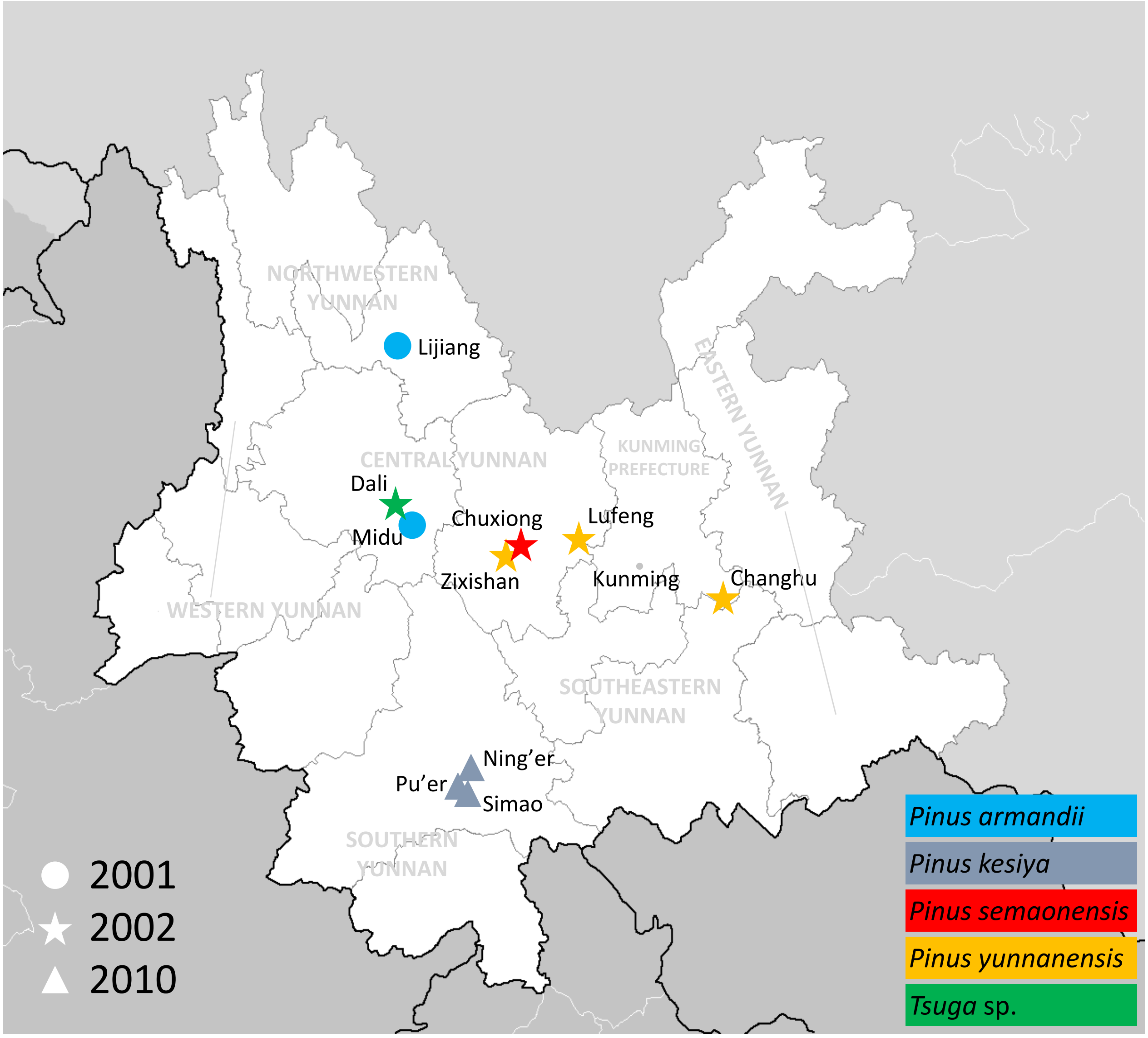

Figure S1. Map of Yunnan Province in China showing the sites and tree hosts from which samples were collected during the three surveys in 2001, 2002 and 2010.

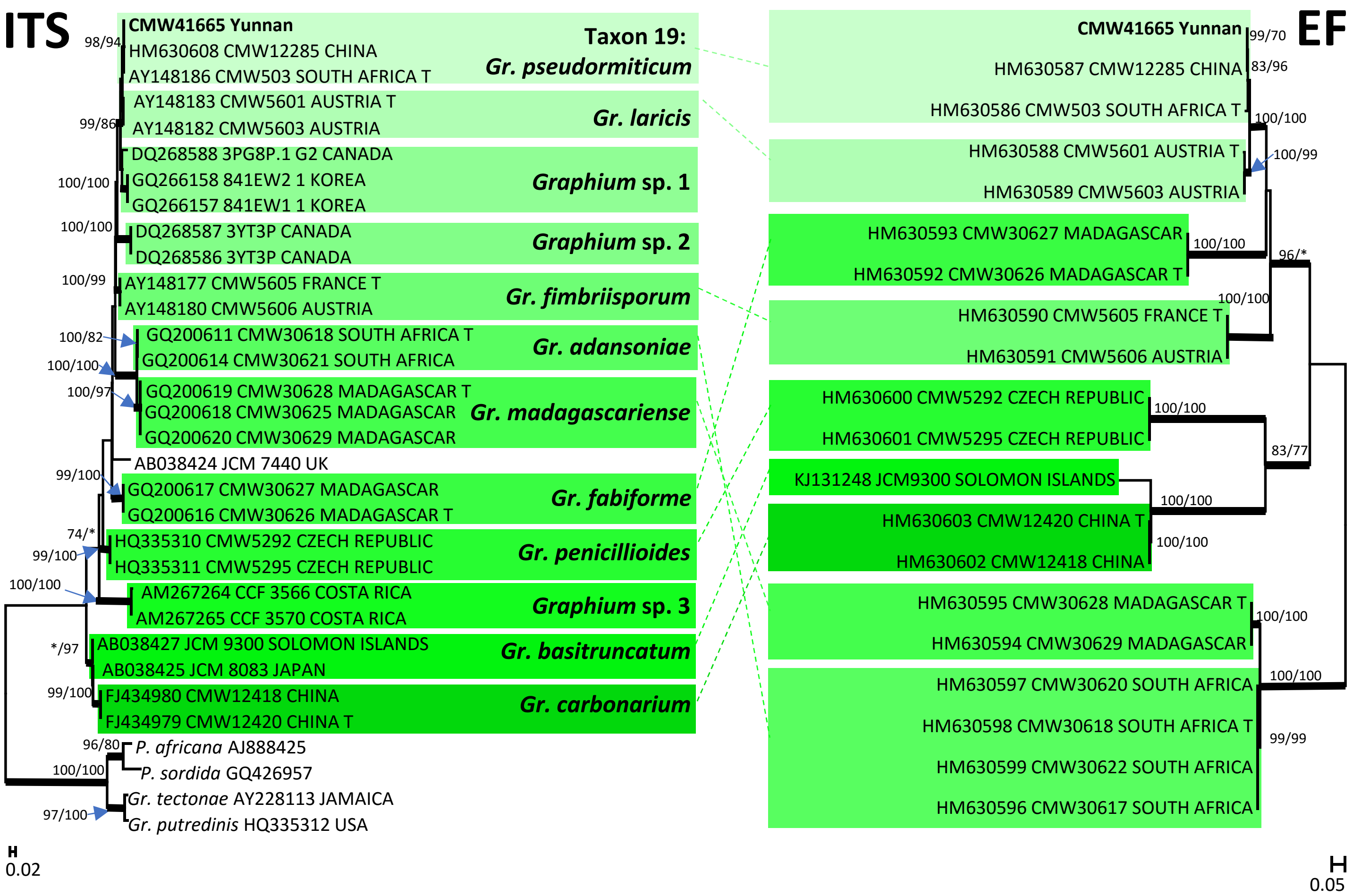

Figure S2. ML trees of the genus *Graphium* generated from DNA sequences of the ITS and BT regions. *Bold* branches indicate posterior probabilities values ≥ 0.95. Bootstrap values ≥ 70 % are recorded at nodes as ML/MP. T = ex-type isolates.

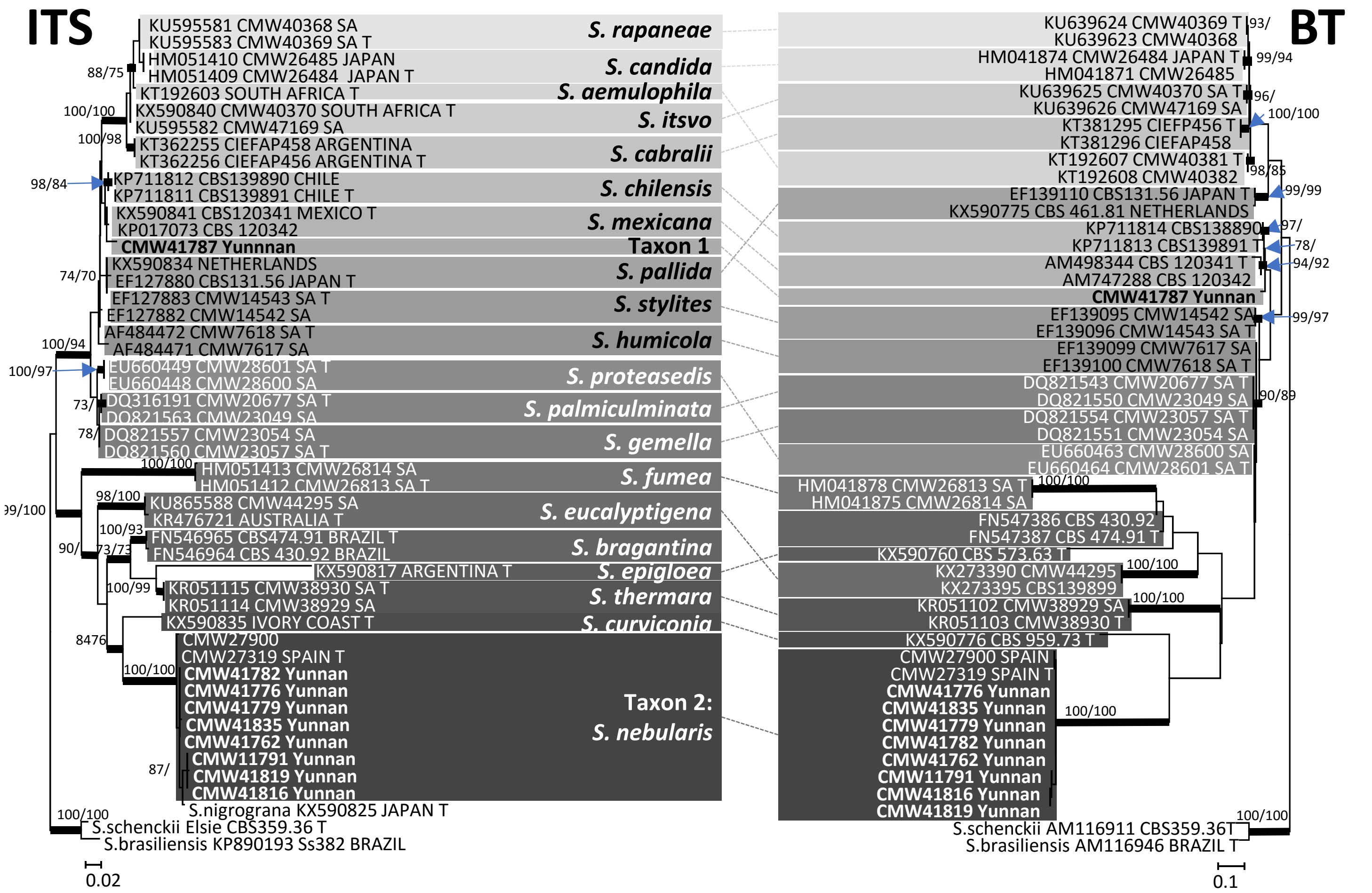

Figure S3. ML trees of the genus *Sporothrix* generated from DNA sequences of ITS and BT regions. *Bold* branches indicate posterior probabilities values ≥ 0.95. Bootstrap values ≥ 70 % are recorded at nodes as ML/MP. T = ex-type isolates.

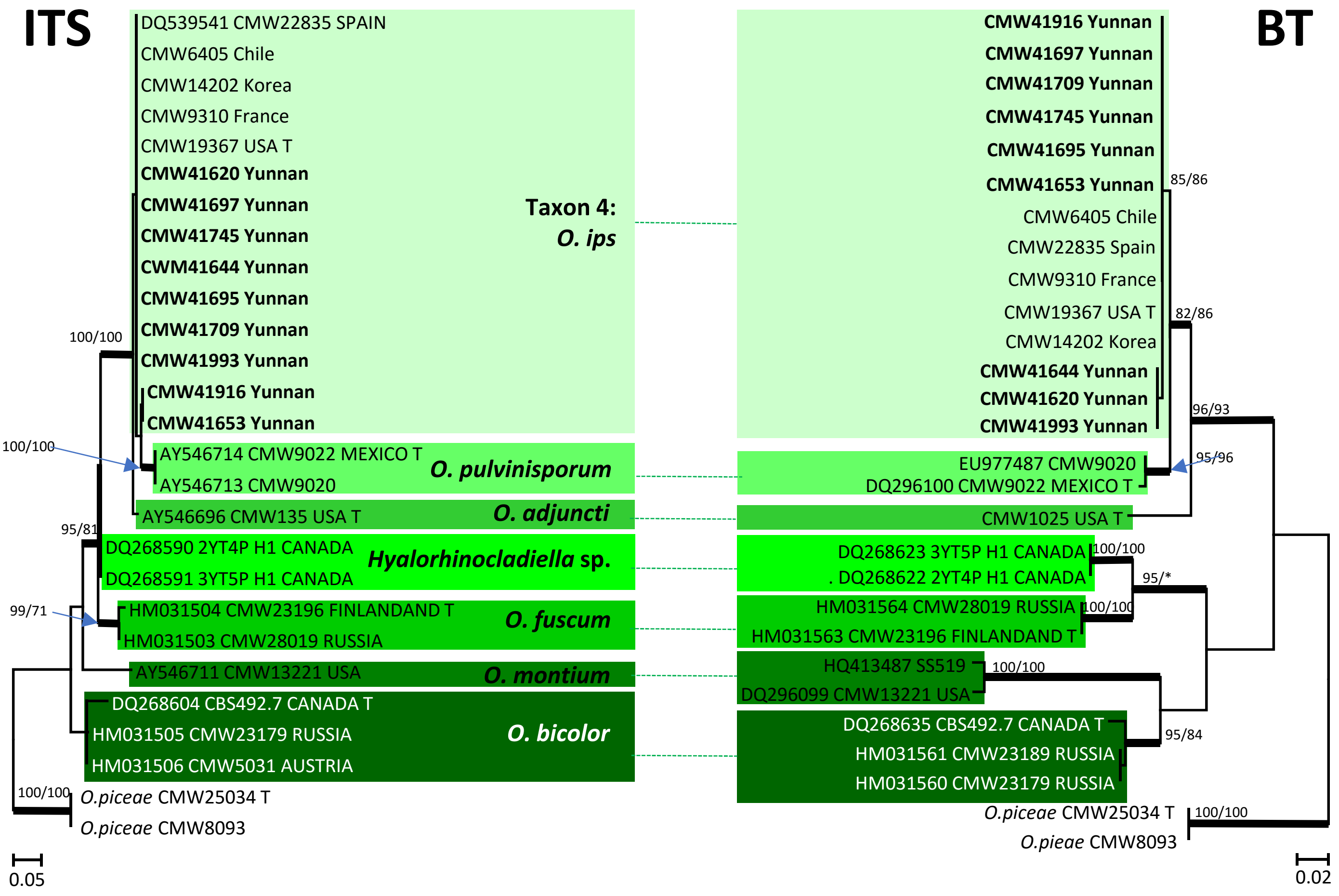

Figure S4. ML trees of the *O. ips* complex generated from DNA sequences of ITS and BT regions. *Bold* branches indicate posterior probabilities values ≥ 0.95. Bootstrap values ≥ 70 % are recorded at nodes as ML/MP. T = ex-type isolates.

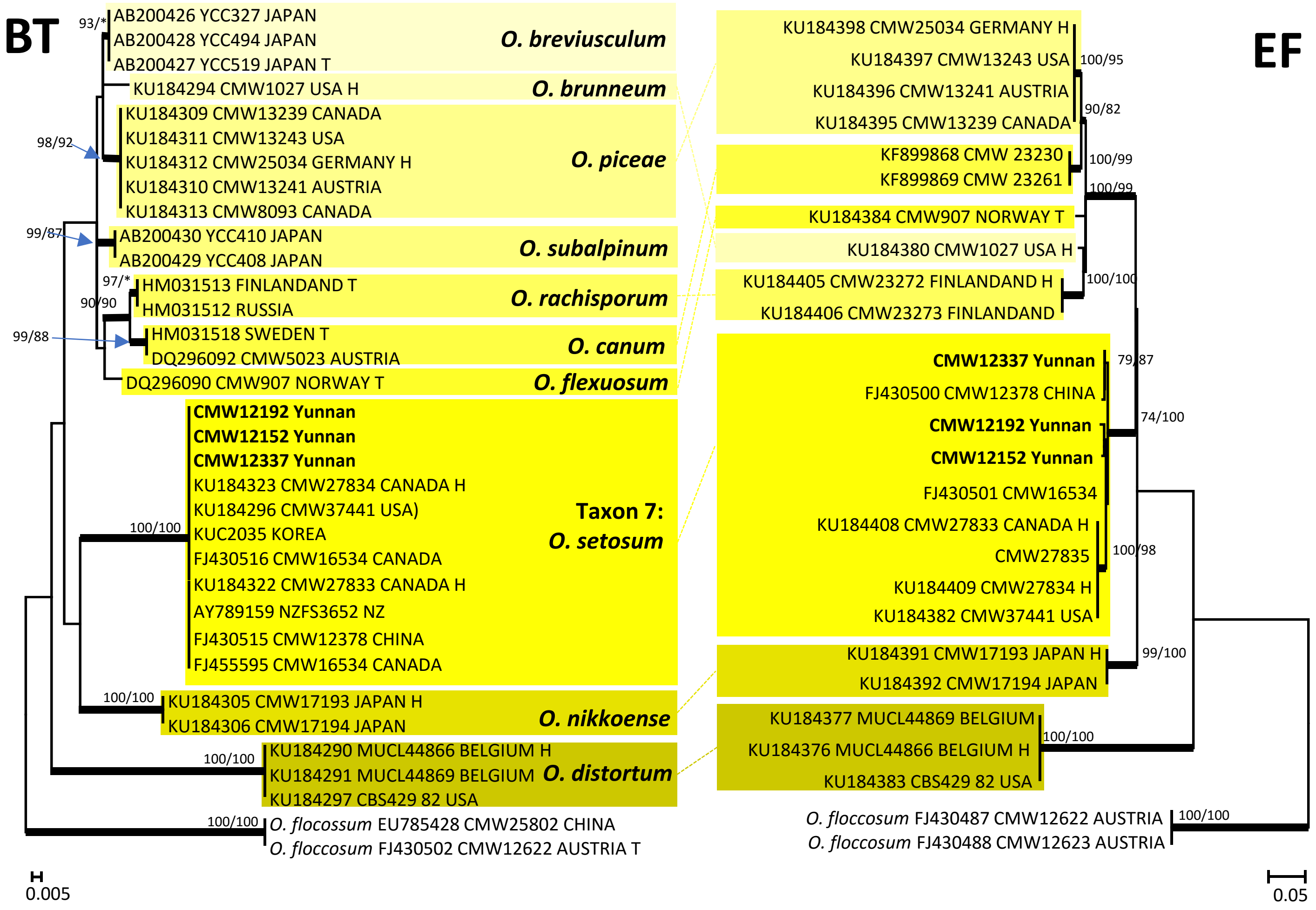

Figure S5. ML trees of the *O. piceae* complex generated from DNA sequences of BT and EF regions. *Bold* branches indicate posterior probabilities values ≥ 0.95. Bootstrap values ≥ 70 % are recorded at nodes as ML/MP. T = ex-type isolates.

Figure S6. ML tree of the *O. quercus* complex generated from DNA sequences of ITS region. *Bold* branches indicate posterior probabilities values ≥ 0.95. Bootstrap values ≥ 70 % are recorded at nodes as ML/MP. T = ex-type isolates.

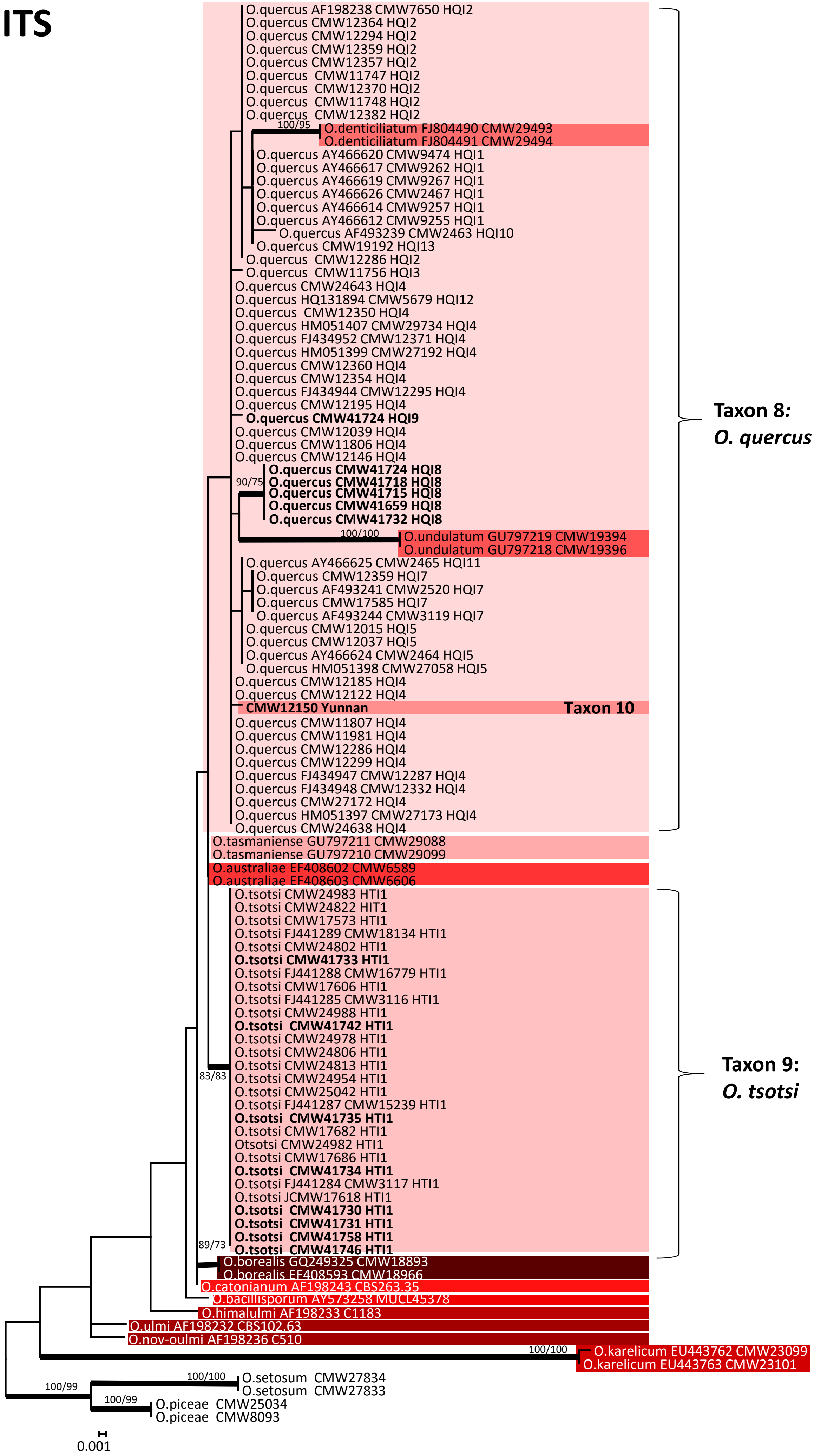

ITS
O.quercus AF198238 CMW7650 HQI2
O.quercus CMW12364 HQI2
O.quercus CMW12294 HQI2
O.quercus CMW12359 HQI2
O.quercus CMW12357 HQI2
O.quercus CMW11747 HQI2
O.quercus CMW12370 HQI2
O.quercus CMW11748 HQI2
O.quercus CMW12382 HQI2
100/95
O.denticiliatum FJ804490 CMW29493
O.denticiliatum FJ804491 CMW29494
O.quercus AY466620 CMW9474 HQI1
O.quercus AY466617 CMW9262 HQI1
O.quercus AY466619 CMW9267 HQI1
O.quercus AY466626 CMW2467 HQI1
O.quercus AY466614 CMW9257 HQI1
O.quercus AY466612 CMW9255 HQI1
O.quercus AF493239 CMW2463 HQI10
O.quercus CMW19192 HQI13
O.quercus CMW12286 HQI2
O.quercus CMW11756 HQI3
O.quercus CMW24643 HQI4
O.quercus HQ131894 CMW5679 HQI12
O.quercus CMW12350 HQI4
O.quercus HM051407 CMW29734 HQI4
O.quercus FJ434952 CMW12371 HQI4
O.quercus HM051399 CMW27192 HQI4
O.quercus CMW12360 HQI4
O.quercus CMW12354 HQI4
O.quercus FJ434944 CMW12295 HQI4
O.quercus CMW12195 HQI4
O.quercus CMW41724 HQI9
O.quercus CMW12039 HQI4
O.quercus CMW11806 HQI4
O.quercus CMW12146 HQI4
O.quercus CMW41724 HQI8
90/75
O.quercus CMW41718 HQI8
O.quercus CMW41715 HQI8
O.quercus CMW41659 HQI8
O.quercus CMW41732 HQI8
100/100
O.undulatum GU797219 CMW19394
O.undulatum GU797218 CMW19396
O.quercus AY466625 CMW2465 HQI11
O.quercus CMW12359 HQI7
O.quercus AF493241 CMW2520 HQI7
O.quercus CMW17585 HQI7
O.quercus AF493244 CMW3119 HQI7
O.quercus CMW12015 HQI5
O.quercus CMW12037 HQI5
O.quercus AY466624 CMW2464 HQI5
O.quercus HM051398 CMW27058 HQI5
O.quercus CMW12185 HQI4
O.quercus CMW12122 HQI4
CMW12150 Yunnan
Taxon 10
O.quercus CMW11807 HQI4
O.quercus CMW11981 HQI4
O.quercus CMW12286 HQI4
O.quercus CMW12299 HQI4
O.quercus FJ434947 CMW12287 HQI4
O.quercus FJ434948 CMW12332 HQI4
O.quercus CMW27172 HQI4
O.quercus HM051397 CMW27173 HQI4
O.quercus CMW24638 HQI4
Taxon 8: O. quercus
O.tasmaniense GU797211 CMW29088
O.tasmaniense GU797210 CMW29099
O.australiae EF408602 CMW6589
O.australiae EF408603 CMW6606
O.tsotsi CMW24983 HTI1
O.tsotsi CMW24822 HIT1
O.tsotsi CMW17573 HTI1
O.tsotsi FJ441289 CMW18134 HTI1
O.tsotsi CMW24802 HTI1
O.tsotsi CMW41733 HTI1
O.tsotsi FJ441288 CMW16779 HTI1
O.tsotsi CMW17606 HTI1
O.tsotsi FJ441285 CMW3116 HTI1
O.tsotsi CMW24988 HTI1
O.tsotsi CMW41742 HTI1
O.tsotsi CMW24978 HTI1
O.tsotsi CMW24806 HTI1
O.tsotsi CMW24813 HTI1
83/83
O.tsotsi CMW24954 HTI1
O.tsotsi CMW25042 HTI1
O.tsotsi FJ441287 CMW15239 HTI1
O.tsotsi CMW41735 HTI1
O.tsotsi CMW17682 HTI1
Otsotsi CMW24982 HTI1
O.tsotsi CMW17686 HTI1
O.tsotsi CMW41734 HTI1
O.tsotsi FJ441284 CMW3117 HTI1
O.tsotsi JCMW17618 HTI1
O.tsotsi CMW41730 HTI1
O.tsotsi CMW41731 HTI1
O.tsotsi CMW41758 HTI1
89/73
O.tsotsi CMW41746 HTI1
Taxon 9: O. tsotsi
O.borealis GQ249325 CMW18893
O.borealis EF408593 CMW18966
O.catonianum AF198243 CBS263.35
O.bacillisporum AY573258 MUCL45378
O.himalulmi AF198233 C1183
O.ulmi AF198232 CBS102.63
O.nov-oulmi AF198236 C510
100/100
O.karelicum EU443762 CMW23099
O.karelicum EU443763 CMW23101
100/100
O.setosum CMW27834
O.setosum CMW27833
100/99
100/99
O.piceae CMW25034
O.piceae CMW8093
0.001

Figure S7. ML tree of the *O. quercus* complex generated from DNA sequences of BT region. *Bold* branches indicate posterior probabilities values ≥ 0.95. Bootstrap values ≥ 70 % are recorded at nodes as ML/MP. T = ex-type isolates.

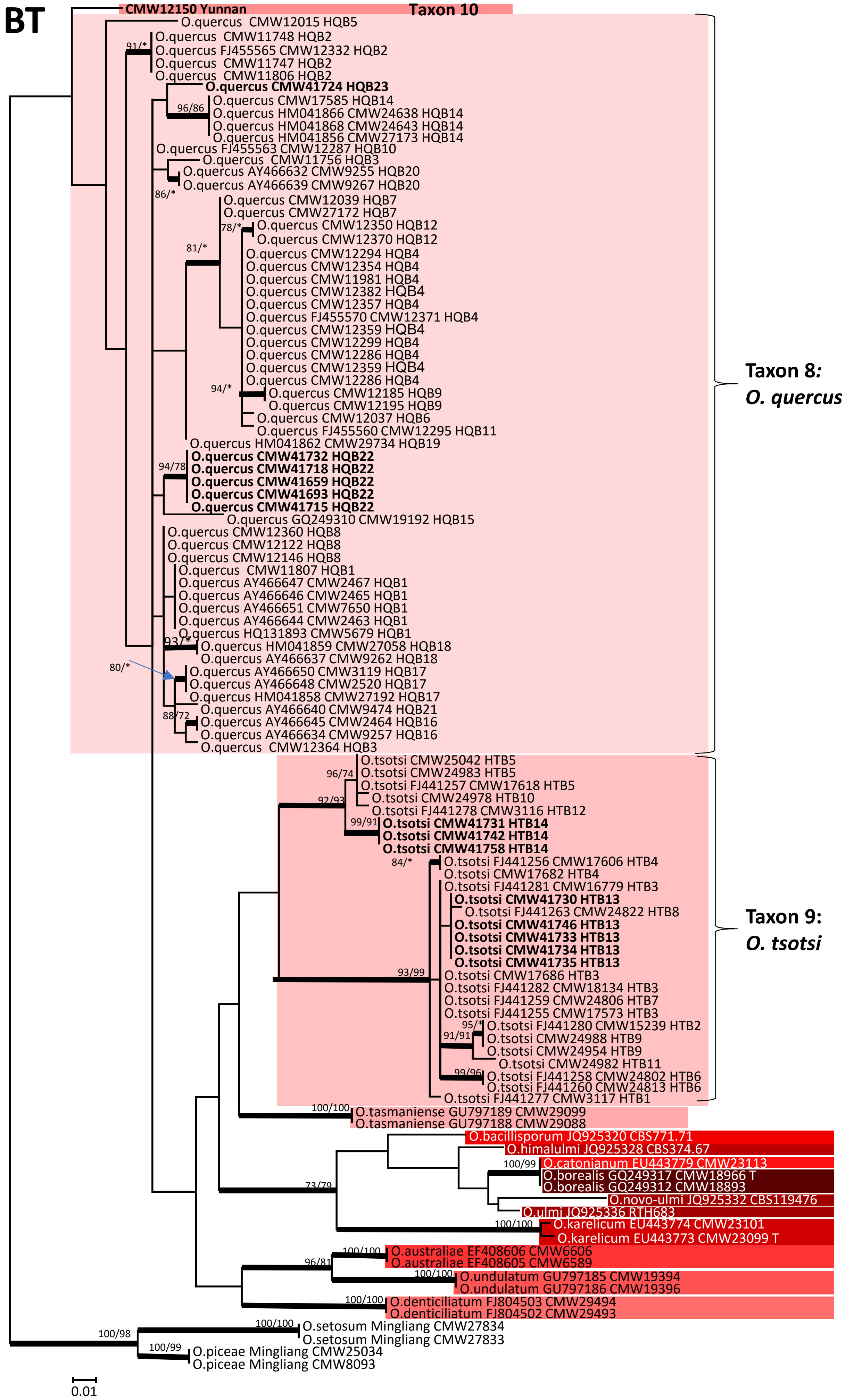
BT
CMW12150 Yunnan
Taxon 10
O.quercus CMW12015 HQB5
O.quercus CMW11748 HQB2
91/*
O.quercus FJ455565 CMW12332 HQB2
O.quercus CMW11747 HQB2
O.quercus CMW11806 HQB2
O.quercus CMW41724 HQB23
O.quercus CMW17585 HQB14
96/86
O.quercus HM041866 CMW24638 HQB14
O.quercus HM041868 CMW24643 HQB14
O.quercus HM041856 CMW27173 HQB14
O.quercus FJ455563 CMW12287 HQB10
O.quercus CMW11756 HQB3
O.quercus AY466632 CMW9255 HQB20
O.quercus AY466639 CMW9267 HQB20
86/*
O.quercus CMW12039 HQB7
O.quercus CMW27172 HQB7
78/*
O.quercus CMW12350 HQB12
O.quercus CMW12370 HQB12
81/*
O.quercus CMW12294 HQB4
O.quercus CMW12354 HQB4
O.quercus CMW11981 HQB4
O.quercus CMW12382 HQB4
O.quercus CMW12357 HQB4
O.quercus FJ455570 CMW12371 HQB4
O.quercus CMW12359 HQB4
O.quercus CMW12299 HQB4
O.quercus CMW12286 HQB4
O.quercus CMW12359 HQB4
O.quercus CMW12286 HQB4
94/*
O.quercus CMW12185 HQB9
O.quercus CMW12195 HQB9
O.quercus CMW12037 HQB6
O.quercus FJ455560 CMW12295 HQB11
O.quercus HM041862 CMW29734 HQB19
94/78
O.quercus CMW41732 HQB22
O.quercus CMW41718 HQB22
O.quercus CMW41659 HQB22
O.quercus CMW41693 HQB22
O.quercus CMW41715 HQB22
O.quercus GQ249310 CMW19192 HQB15
O.quercus CMW12360 HQB8
O.quercus CMW12122 HQB8
O.quercus CMW12146 HQB8
O.quercus CMW11807 HQB1
O.quercus AY466647 CMW2467 HQB1
O.quercus AY466646 CMW2465 HQB1
O.quercus AY466651 CMW7650 HQB1
O.quercus AY466644 CMW2463 HQB1
O.quercus HQ131893 CMW5679 HQB1
93/*
O.quercus HM041859 CMW27058 HQB18
O.quercus AY466637 CMW9262 HQB18
80/*
O.quercus AY466650 CMW3119 HQB17
O.quercus AY466648 CMW2520 HQB17
O.quercus HM041858 CMW27192 HQB17
88/72
O.quercus AY466640 CMW9474 HQB21
O.quercus AY466645 CMW2464 HQB16
O.quercus AY466634 CMW9257 HQB16
O.quercus CMW12364 HQB3
Taxon 8: O. quercus
O.tsotsi CMW25042 HTB5
96/74
O.tsotsi CMW24983 HTB5
O.tsotsi FJ441257 CMW17618 HTB5
92/93
O.tsotsi CMW24978 HTB10
O.tsotsi FJ441278 CMW3116 HTB12
99/91
O.tsotsi CMW41731 HTB14
O.tsotsi CMW41742 HTB14
O.tsotsi CMW41758 HTB14
84/*
O.tsotsi FJ441256 CMW17606 HTB4
O.tsotsi CMW17682 HTB4
O.tsotsi FJ441281 CMW16779 HTB3
O.tsotsi CMW41730 HTB13
O.tsotsi FJ441263 CMW24822 HTB8
O.tsotsi CMW41746 HTB13
O.tsotsi CMW41733 HTB13
O.tsotsi CMW41734 HTB13
O.tsotsi CMW41735 HTB13
93/99
O.tsotsi CMW17686 HTB3
O.tsotsi FJ441282 CMW18134 HTB3
O.tsotsi FJ441259 CMW24806 HTB7
O.tsotsi FJ441255 CMW17573 HTB3
95/*
O.tsotsi FJ441280 CMW15239 HTB2
91/91
O.tsotsi CMW24988 HTB9
O.tsotsi CMW24954 HTB9
O.tsotsi CMW24982 HTB11
99/96
O.tsotsi FJ441258 CMW24802 HTB6
O.tsotsi FJ441260 CMW24813 HTB6
O.tsotsi FJ441277 CMW3117 HTB1
Taxon 9: O. tsotsi
100/100
O.tasmaniense GU797189 CMW29099
O.tasmaniense GU797188 CMW29088
O.bacillisporum JQ925320 CBS771.71
O.himalulmi JQ925328 CBS374.67
100/99
O.catonianum EU443779 CMW23113
O.borealis GQ249317 CMW18966 T
O.borealis GQ249312 CMW18893
73/79
O.novo-ulmi JQ925332 CBS119476
O.ulmi JQ925336 RTH683
100/100
O.karelicum EU443774 CMW23101
O.karelicum EU443773 CMW23099 T
100/100
O.australiae EF408606 CMW6606
96/81
O.australiae EF408605 CMW6589
100/100
O.undulatum GU797185 CMW19394
O.undulatum GU797186 CMW19396
100/100
O.denticiliatum FJ804503 CMW29494
O.denticiliatum FJ804502 CMW29493
100/100
O.setosum Mingliang CMW27834
100/98
O.setosum Mingliang CMW27833
100/99
O.piceae Mingliang CMW25034
O.piceae Mingliang CMW8093
0.01

Figure S8. ML tree of the *O. quercus* complex generated from DNA sequences of EF region. *Bold* branches indicate posterior probabilities values ≥ 0.95. Bootstrap values ≥ 70 % are recorded at nodes as ML/MP. T = ex-type isolates.

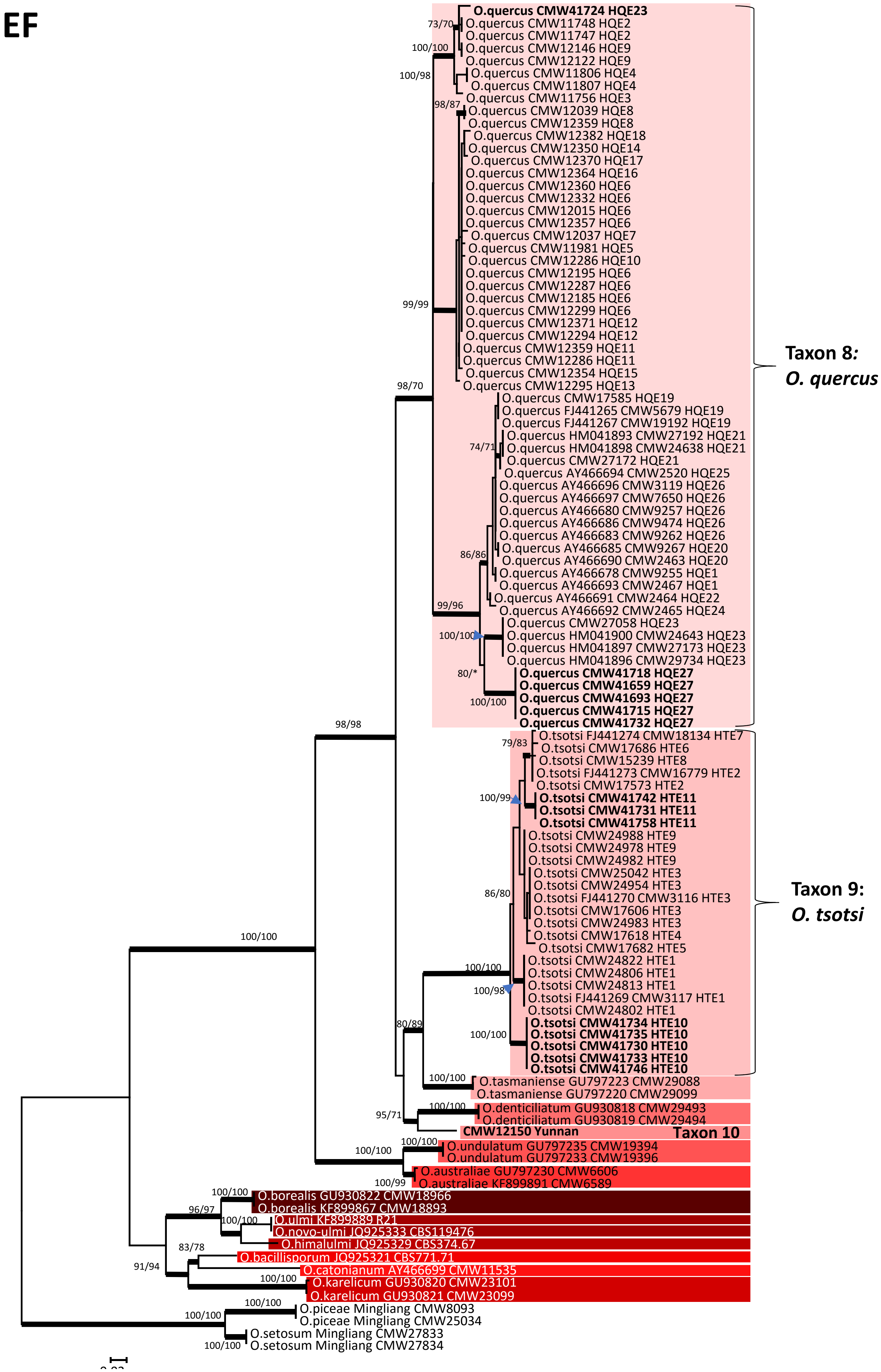

EF
O.quercus CMW41724 HQE23
73/70
O.quercus CMW11748 HQE2
O.quercus CMW11747 HQE2
100/100
O.quercus CMW12146 HQE9
O.quercus CMW12122 HQE9
100/98
O.quercus CMW11806 HQE4
O.quercus CMW11807 HQE4
O.quercus CMW11756 HQE3
98/87
O.quercus CMW12039 HQE8
O.quercus CMW12359 HQE8
O.quercus CMW12382 HQE18
O.quercus CMW12350 HQE14
O.quercus CMW12370 HQE17
O.quercus CMW12364 HQE16
O.quercus CMW12360 HQE6
O.quercus CMW12332 HQE6
O.quercus CMW12015 HQE6
O.quercus CMW12357 HQE6
O.quercus CMW12037 HQE7
O.quercus CMW11981 HQE5
O.quercus CMW12286 HQE10
O.quercus CMW12195 HQE6
O.quercus CMW12287 HQE6
O.quercus CMW12185 HQE6
99/99
O.quercus CMW12299 HQE6
O.quercus CMW12371 HQE12
O.quercus CMW12294 HQE12
O.quercus CMW12359 HQE11
O.quercus CMW12286 HQE11
O.quercus CMW12354 HQE15
98/70
O.quercus CMW12295 HQE13
O.quercus CMW17585 HQE19
O.quercus FJ441265 CMW5679 HQE19
O.quercus FJ441267 CMW19192 HQE19
O.quercus HM041893 CMW27192 HQE21
74/71
O.quercus HM041898 CMW24638 HQE21
O.quercus CMW27172 HQE21
O.quercus AY466694 CMW2520 HQE25
O.quercus AY466696 CMW3119 HQE26
O.quercus AY466697 CMW7650 HQE26
O.quercus AY466680 CMW9257 HQE26
O.quercus AY466686 CMW9474 HQE26
O.quercus AY466683 CMW9262 HQE26
O.quercus AY466685 CMW9267 HQE20
86/86
O.quercus AY466690 CMW2463 HQE20
O.quercus AY466678 CMW9255 HQE1
O.quercus AY466693 CMW2467 HQE1
O.quercus AY466691 CMW2464 HQE22
99/96
O.quercus AY466692 CMW2465 HQE24
O.quercus CMW27058 HQE23
100/100
O.quercus HM041900 CMW24643 HQE23
O.quercus HM041897 CMW27173 HQE23
O.quercus HM041896 CMW29734 HQE23
80/*
O.quercus CMW41718 HQE27
O.quercus CMW41659 HQE27
100/100
O.quercus CMW41693 HQE27
O.quercus CMW41715 HQE27
O.quercus CMW41732 HQE27
Taxon 8:
O. quercus
98/98
O.tsotsi FJ441274 CMW18134 HTE7
79/83
O.tsotsi CMW17686 HTE6
O.tsotsi CMW15239 HTE8
O.tsotsi FJ441273 CMW16779 HTE2
O.tsotsi CMW17573 HTE2
100/99
O.tsotsi CMW41742 HTE11
O.tsotsi CMW41731 HTE11
O.tsotsi CMW41758 HTE11
O.tsotsi CMW24988 HTE9
O.tsotsi CMW24978 HTE9
O.tsotsi CMW24982 HTE9
O.tsotsi CMW25042 HTE3
O.tsotsi CMW24954 HTE3
86/80
O.tsotsi FJ441270 CMW3116 HTE3
O.tsotsi CMW17606 HTE3
O.tsotsi CMW24983 HTE3
O.tsotsi CMW17618 HTE4
O.tsotsi CMW17682 HTE5
100/100
O.tsotsi CMW24822 HTE1
O.tsotsi CMW24806 HTE1
O.tsotsi CMW24813 HTE1
100/98
O.tsotsi FJ441269 CMW3117 HTE1
O.tsotsi CMW24802 HTE1
80/89
O.tsotsi CMW41734 HTE10
100/100
O.tsotsi CMW41735 HTE10
O.tsotsi CMW41730 HTE10
O.tsotsi CMW41733 HTE10
O.tsotsi CMW41746 HTE10
Taxon 9:
O. tsotsi
100/100
O.tasmaniense GU797223 CMW29088
O.tasmaniense GU797220 CMW29099
100/100
100/100
O.denticiliatum GU930818 CMW29493
95/71
O.denticiliatum GU930819 CMW29494
CMW12150 Yunnan
Taxon 10
100/100
O.undulatum GU797235 CMW19394
100/100
O.undulatum GU797233 CMW19396
O.australiae GU797230 CMW6606
100/99
O.australiae KF899891 CMW6589
100/100
O.borealis GU930822 CMW18966
96/97
O.borealis KF899867 CMW18893
100/100
O.ulmi KF899889 R21
O.novo-ulmi JQ925333 CBS119476
83/78
O.himalulmi JQ925329 CBS374.67
O.bacillisporum JQ925321 CBS771.71
91/94
O.catonianum AY466699 CMW11535
100/100
O.karelicum GU930820 CMW23101
O.karelicum GU930821 CMW23099
100/100
O.piceae Mingliang CMW8093
100/100
O.piceae Mingliang CMW25034
O.setosum Mingliang CMW27833
100/100
O.setosum Mingliang CMW27834

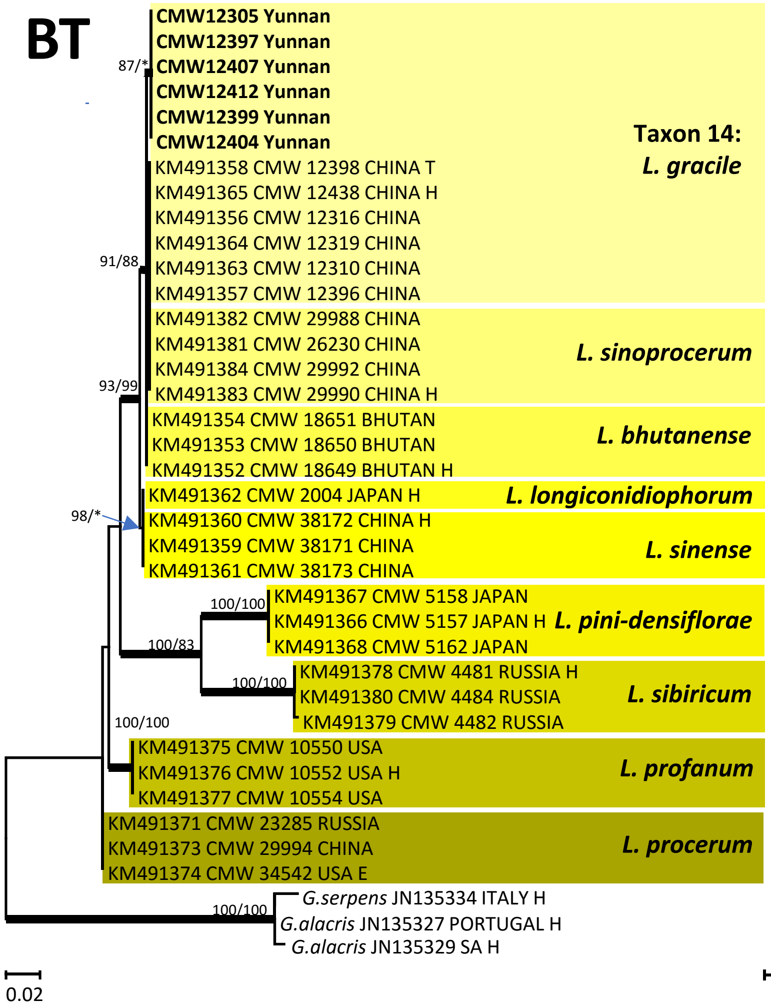

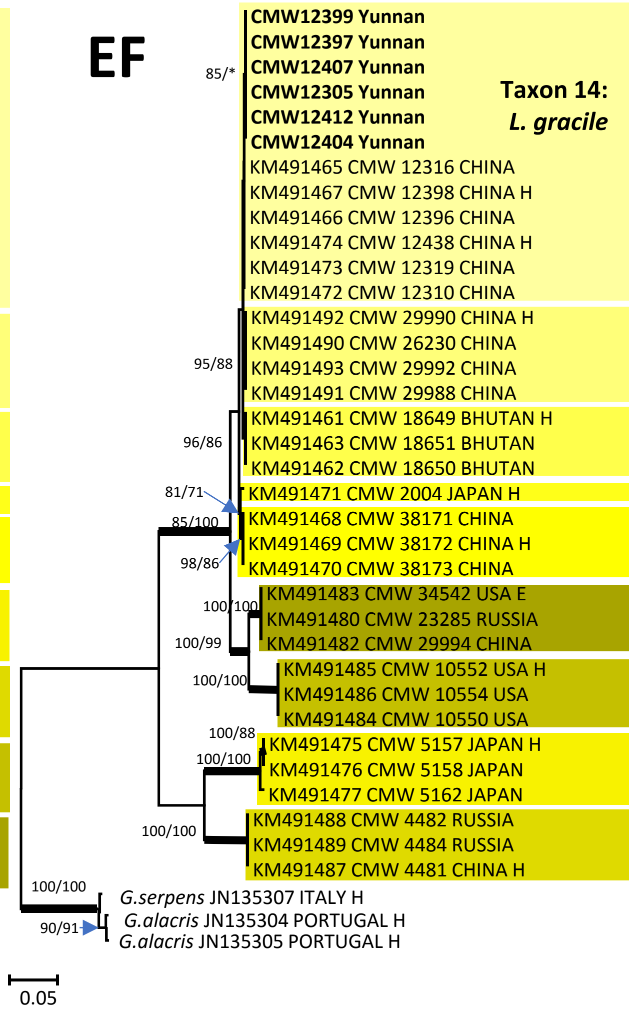

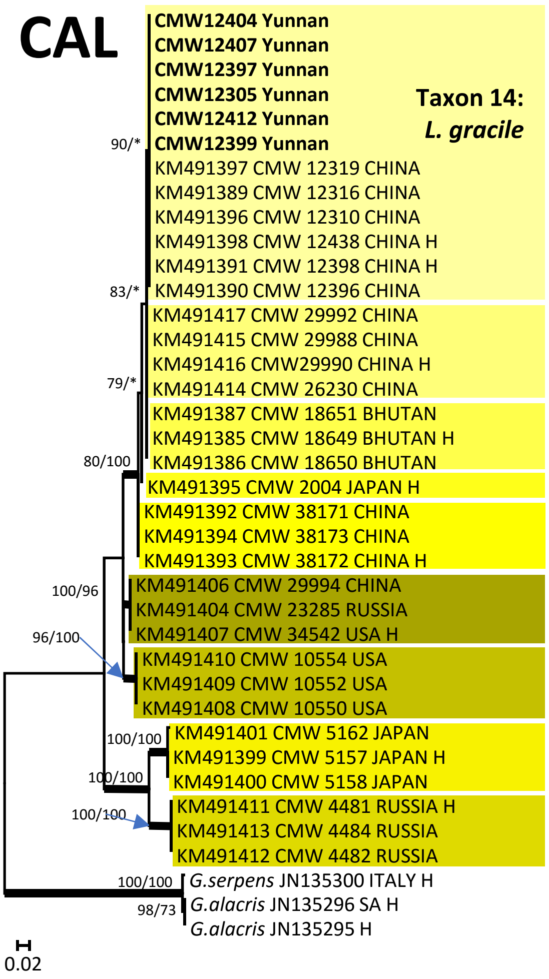

Figure S9. ML trees of the *L. procerum* complex generated from DNA sequences of BT, EF and CAL regions. *Bold* branches indicate posterior probabilities values ≥ 0.95. Bootstrap values ≥ 70 % are recorded at nodes as ML/MP. T = ex-type isolates.

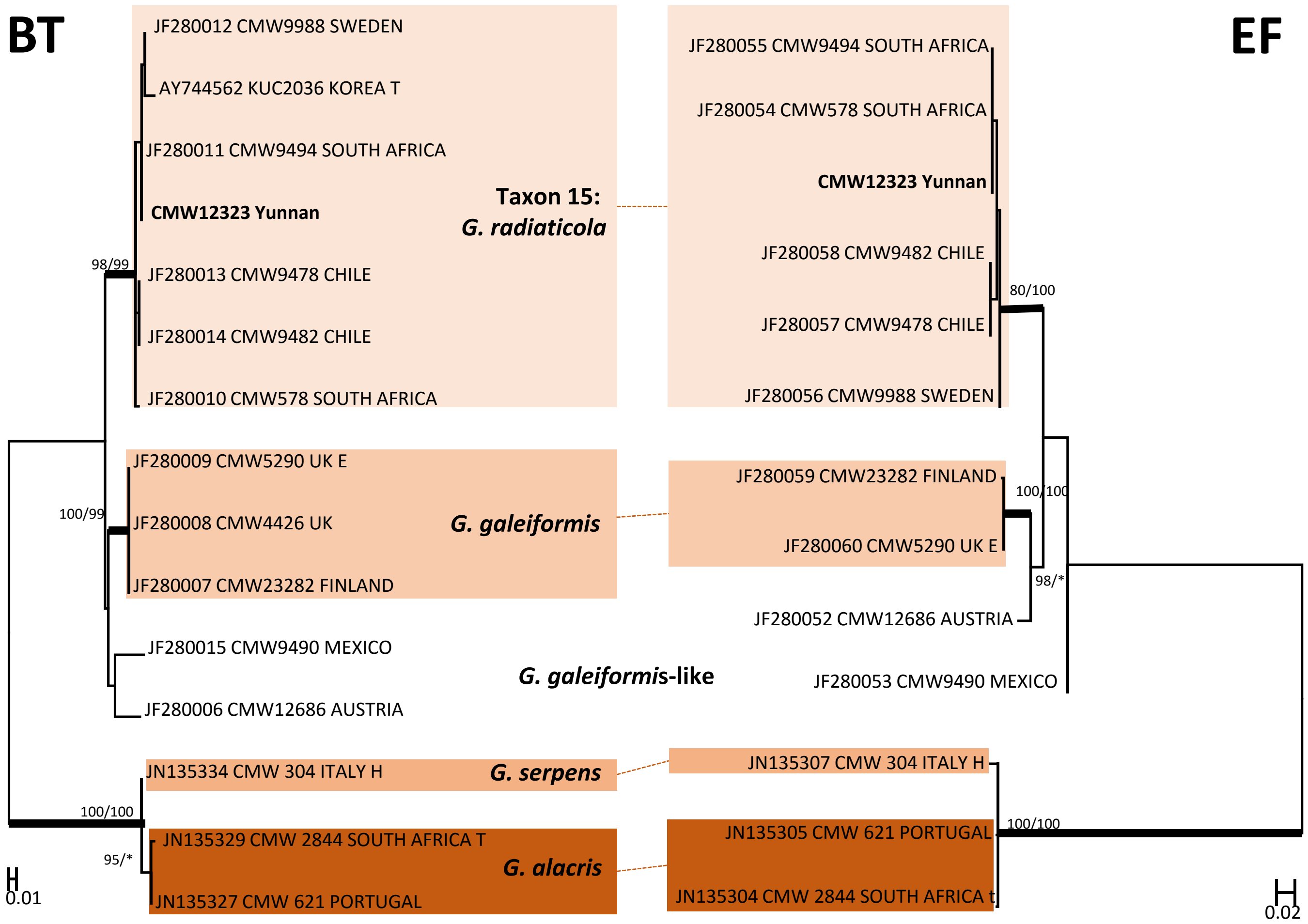

Figure S10. ML trees of the *G. galeiformis* complex generated from DNA sequences of BT and EF regions. *Bold* branches indicate posterior probabilities values ≥ 0.95. Bootstrap values ≥ 70 % are recorded at nodes as ML/MP. T = ex-type isolates.

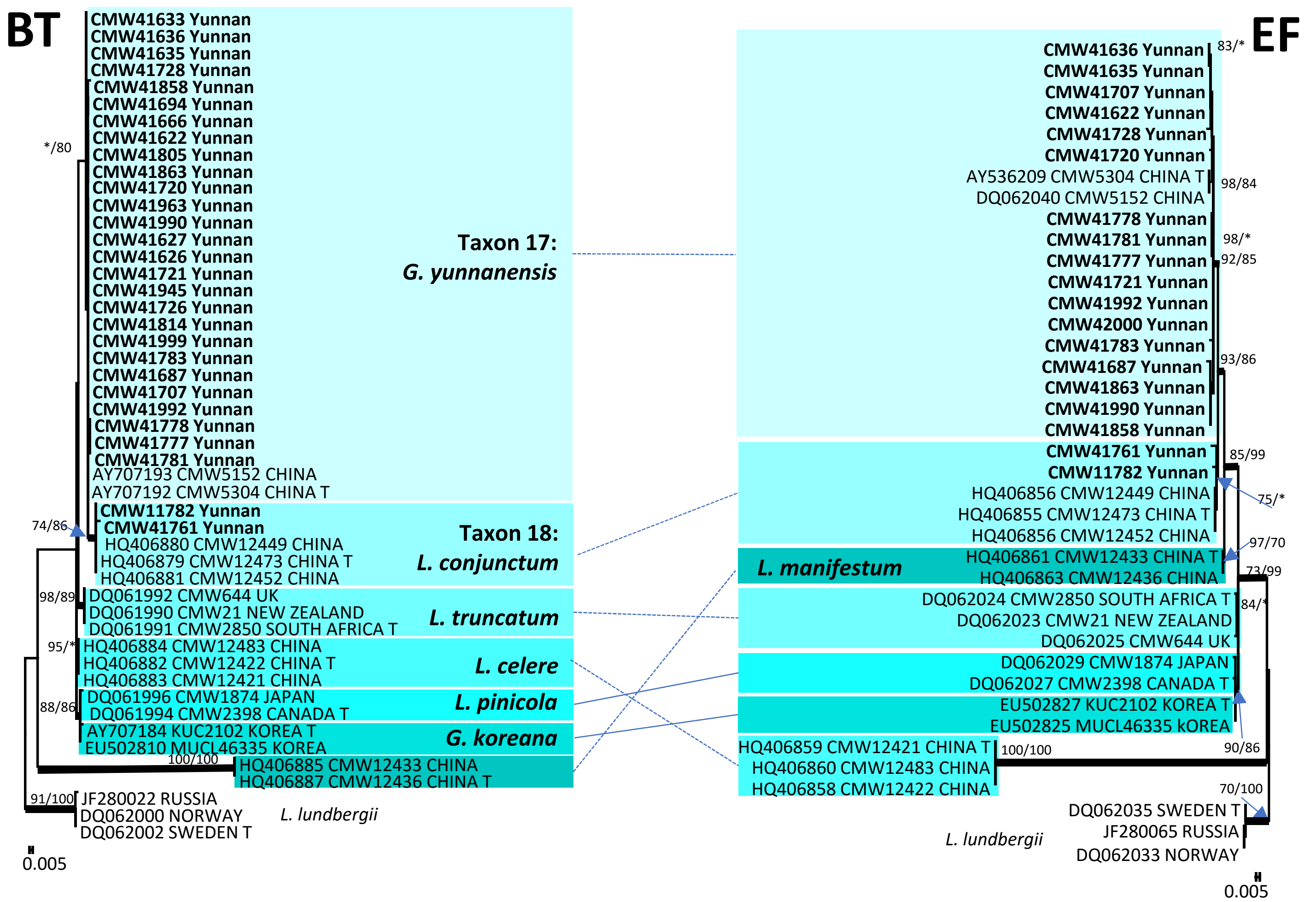

Figure S11. ML trees of the *L. lundbergii* complex generated from DNA sequences of BT and EF regions. *Bold* branches indicate posterior probabilities values ≥ 0.95. Bootstrap values ≥ 70 % are recorded at nodes as ML/MP. T = ex-type isolates.
